# Enhancing decision-making in glioblastoma surgery through an explainable human-AI collaboration: an international multicenter model development and external validation study

**DOI:** 10.1038/s41698-025-01183-2

**Published:** 2025-11-27

**Authors:** Julius M. Kernbach, Urte Schroeder, Karlijn Hakvoort, Jonas Ort, Hussam Hamou, Danilo Bzdok, Yasin Temel, Pieter Kubben, Charlotte Weyland, Martin Wiesmann, Victor Staartjes, Kevin Akeret, Moira Vieli, Carlo Serra, Luca Regli, Stefan Grau, Lasse Dührsen, Franz Ricklefs, Oliver Schnell, David Ryan Ormond, Alexander Grote, Matthias Simon, Hagen Meredig, Marianne Schell, Martin Bendszus, Georg Neuloh, Hans Clusmann, Dieter-Henrik Heiland, Daniel Delev

**Affiliations:** 1 Department of Neuroradiology, Heidelberg University, Heidelberg, Germany; 2 Neurosurgical Artificial Intelligence Laboratory Aachen (NAILA), RWTH Aachen University Hospital, Aachen, Germany; 3 Department of Neurosurgery, RWTH Aachen University Hospital, Aachen, Germany; 4 Mila - Quebec Artificial Intelligence Institute, Montreal, QC Canada; 5 The Neuro - Montreal Neurological Institute (MNI), Department of Biomedical Engineering, McGill University, Montreal, QC Canada; 6 Department of Neurosurgery, Maastricht University Medical Center + , 6229 HX Maastricht, The Netherlands; 7 Department of Neuroradiology, RWTH Aachen University Hospital, Aachen, Germany; 8 Machine Intelligence in Clinical Neuroscience (MICN) Laboratory, Department of Neurosurgery, Clinical Neuroscience Center, University Hospital Zurich, University of Zurich, Zurich, Switzerland; 9 Department of Neurosurgery, University Hospital Zurich, University of Zurich, Zurich, Switzerland; 10 Department of Neurosurgery, University of Cologne, Cologne, Germany; 11 Department of Neurosurgery, University of Marburg, Campus, Fulda Germany; 12 Department of Neurosurgery, University Medical Center Hamburg-Eppendorf, Hamburg, Germany; 13 Department of Neurosurgery, University of Erlangen, Erlangen, Germany; 14 Department of Neurosurgery, University of Colorado Anschutz Medical Campus, Aurora, CO USA; 15 Clinic for Neurosurgery, Philipps University of Marburg, Marburg, Germany; 16 Department of Neurosurgery, Evangelisches Klinikum Bethel, Universitätsklinikum OWL, Bielefeld, Germany; 17 Department of Neurosurgery, Bremerhaven-Reinkenheide Hospital, Bremerhaven, Germany

**Keywords:** Surgical oncology, CNS cancer

## Abstract

Surgical resection improves survival in glioblastoma, yet predicting the extent of resection (EOR) remains highly challenging. We developed and externally validated an explainable AI model to generate personalized EOR estimates in 811 glioblastoma patients undergoing microsurgical resection. EOR was categorized into gross-total (GTR), near-total (NTR), and subtotal resections (STR). An interpretable framework provided model explanations and sensitivity analyses to assess the model’s strengths and limitations. To demonstrate clinical impact, we compared the performance of the human expert (gold standard) with our AI model and a combined *human-AI* approach. External validation confirmed generalizability (AUC 0.78, CI 0.73-0.82). Class-specific AUCs were 0.75 (0.67-0.82) for GTR, 0.59 (0.50-0.69) for NTR, and 0.69 (0.53-0.85) for STR. Key predictors included KPS and NANO scores, age, tumor volume, and unfavorable anatomical locations. A combined human-AI collaboration outperformed human experts, with higher overall accuracies (0.53 to 0.94), F1 scores (0.30 to 0.92), and Cohen’s κ (0.41 to 0.84). Enhancing predictive performance through the clinician-AI collaboration, our explainable model supports preoperative planning and highlights the value of integrating machine intelligence into surgical decision-making.

## Introduction

Glioblastoma (GBM), the most prevalent and deadliest malignant brain tumor, inflicts permanent injury to the brain and shows high resistance to even the most advanced treatment options^1,2^. Median survival ranges from 12 to 15 months, with a 5-year survival rate of only ~5%^2,3^. Tumor progression is inevitable and leads to severe disability, preventing independent functioning and severely reducing the quality of life^4–6^.

Among available treatments, surgical resection, when possible, is of highest importance for GBM management. Achieving a gross total resection (GTR), defined as the complete removal of the contrast-enhancing tumor, is a key determinant of improved survival^7,8^. However, aggressive resection increases the risk of neurological deterioration, limiting treatment options and adversely affecting overall survival. Therefore, the standard surgical approach involves maximizing tumor removal while preserving neurological function, commonly referred to as maximal-safe resection^7,9^. Recent evidence indicates that the survival benefit of EOR varies across epigenetic subtypes of GBM, underscoring the importance of tailoring surgical strategies to the individual^10^. As surgery becomes increasingly guided by molecular profiles, the precise preoperative assessment of EOR feasibility and risk is critical.

Yet, the inherently personalized nature of surgical decision-making, influenced by anatomical heterogeneity and proximity to eloquent brain regions, among other factors, renders an accurate prediction of EOR exceptionally challenging^6,7^. Among patients deemed eligible for maximal-safe resection and anticipated to achieve GTR, fewer than 25% ultimately do so^11^. Estimations of EOR by experienced neuro-oncologic surgeons remain imprecise, often marked by overconfidence, bias, lack of uniformity, and substantial variability among different neurosurgeons^11–14^. As such, predicting and achieving the optimal EOR represent the most uncertain and individualized step in GBM treatment.

The inherent complexity highlights the urgent need for robust, personalized prediction tools. Early efforts to address this through machine learning have been hindered by methodological flaws, including small sample sizes, opaque architectures, and the absence of external validation^15^. Often, these models also function as “black boxes,” generating predictions without revealing how input features contribute to the outcome, thereby limiting clinical trust and interpretability^16^.

To address these limitations, we developed and externally validated an explainable AI model that generates personalized preoperative risk estimates for EOR in newly diagnosed GBM patients. Our approach emphasizes interpretability, transparency, and clinical utility. Specifically, we leveraged SHapley Additive exPlanations (SHAP) to deconstruct model predictions across different levels of EOR, enabling both global and patient-level insights into feature contributions. Such interpretability is particularly important in multiclass settings, where understanding class-specific drivers can help clinicians distinguish between competing outcomes in borderline cases. Understanding why the model favors one surgical outcome over another further enhances trust and clinical usability. Rather than replacing clinical judgment, our model supports a collaborative framework that enables clinicians to explore and contextualize AI-derived predictions. This human-AI synergy aims to refine surgical planning and promote more personalized, data-driven decision-making in neuro-oncology.

## Results

### Model development and internal validation

**Fig. 1:**
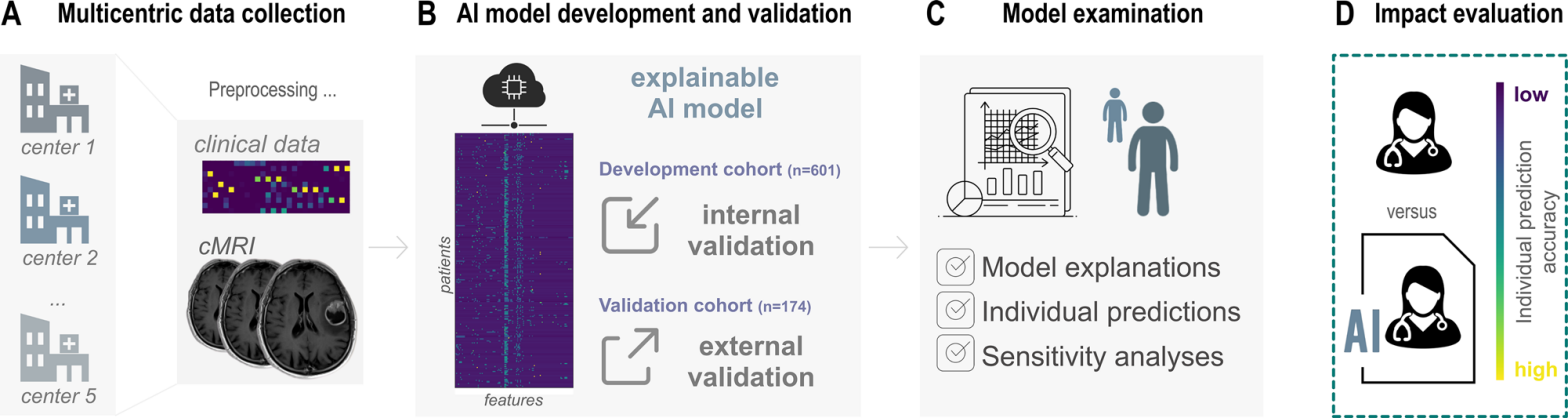
Concept overview of the explainable AI approach. Adhering to established AI reporting guidelines^19,24,69^, our approach creates an accessible, transparent AI application with an emphasis on *interpretability* and *explainability*. **A** Five international centers provided data for model development and internal validation. **B** The explainable AI model was developed using strict machine learning standards^27,69–71^ and evaluated in a set-aside internal validation dataset. The final AI model was brought forward to a held-out external cohort from a sixth center, providing the highest level of validation^31,72^. **C** We empowered every clinician to gain a deeper understanding of how the AI model works using different techniques for model examination. First, we provided *global* explanations of how our algorithm derived accurate class predictions. Secondly, we visualized *local*, that is, individual predictions for specific patients. Thirdly, we conducted sensitivity analyses to demonstrate the algorithm’s performance under extreme conditions and to highlight situations that require heightened vigilance in interpreting the model’s outcome. **D** To evaluate the real-life clinical impact and benefit, we compared the performance of the AI model against the current gold standard, the expert physician’s estimation of EOR. Lastly, we combined the strengths of the algorithm and the physician to propose a synergistic collaboration between clinicians and AI, enabling even more informed and precise clinical decision-making.

**Fig. 2:**
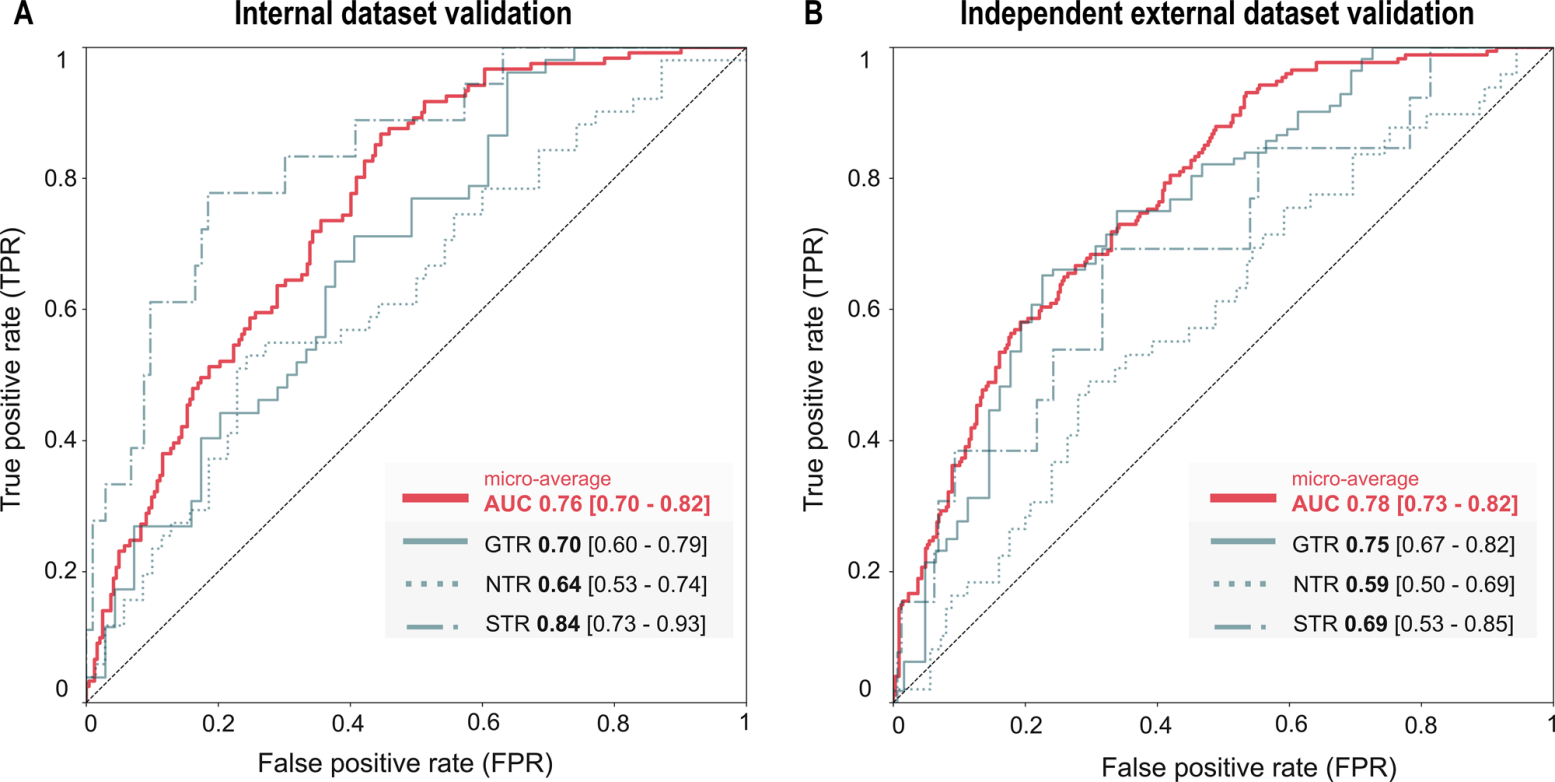
Internal and external validated model performance. Predictive model performance is demonstrated using receiver operating characteristics (ROC) curves in (**A**) internal and (**B**) external validation cohorts. The red curve represents the micro-averaged ROC, which aggregates the contributions of all three classes—gross-total (GTR), near-total (NTR), and subtotal resection (STR), by treating each class prediction as an individual binary classification. This approach provides a single overall AUC score that reflects the model’s ability to distinguish between all class labels, weighted by class frequency. The AUC reached 0.76 in the internal and 0.78 in the external validation, indicating consistent and robust performance across datasets. As the most rigorous test of model generalizability, external validation confirmed the model’s applicability to different populations^31,72^. Class-specific one-versus-rest predictions (shown in shades of turquoise) revealed the highest discrimination for STR and GTR. At the same time, performance for NTR was more limited, reflecting the inherent difficulty in predicting borderline resections.

We developed a machine-learning model in a cohort of 601 patients with newly (Fig. 1) diagnosed GBM (Table 1 and Supplementary Table 3 for details). Several supervised algorithms adapted to the multiclass setting were assessed during model selection (Supplementary Table 1). In a data-driven fashion, the most competitive model, a random forest classifier^17^, was selected, all models’ parameters held fixed, and then applied to the held-out validation data without further parameter adjustment. The AI model resulted in an AUC of 0.76 (95% confidence intervals (CI) 0.70 - 0.82) micro-averaged across all EOR categories (Fig. 2A). Discrimination performance for predicting GTR resulted in an AUC of 0.70 (CI 0.60 - 0.79), with precision and recall of 0.66 (CI 0.57 - 0.74) and 0.64 (CI 0.55 - 0.72). Estimations of NTR amounted to an AUC of 0.64 (CI 0.53 - 0.74), a precision of 0.59 (CI 0.50 - 0.68), and a recall of 0.56 (CI 0.46 - 0.65). Predictions for the STR class resulted in an AUC of 0.84 (CI 0.73 - 0.93), a precision of 0.83 (CI 0.75 - 0.90), and a recall of 0.84 (CI 0.77 - 0.90). Calibration curves aligned well with the internal dataset’s ideal reference line, supporting reliable probability estimates across all EOR classes (Supplementary Fig. 1A), which was supported by Brier scores of 0.30 (GTR), 0.28 (NTR), and 0.44 (STR), reflecting fair overall agreement between predicted and actual probabilities.

**Table 1:** Characteristics of the development and validation cohort

		Cohort	
	Feature	Development	External Validation
		(*n* = 601)	(*n* = 174)
**Clinical Information**	Female sex	237 (39.4)	78 (44.9)
	No. missing	0	0
	Age	62.2 ± 11.9	62.4 ± 12.7
	No. missing	0	3
	Preoperative NANO score	2.2 ± 1.9	2.1 ± 2.3
	No. missing	3	3
	Preoperative KPS score	78.8 ± 16.1	83.3 ± 16.0
	No. missing	57	0
	Epilepsy	163 (27.2)	56 (32.6)
	No. missing	2	2
**Tumor Topography and Location**	Tumor volume	48.6 ± 83.4	34.0 ± 28.9
	No. missing	57	0
	Eloquence	275 (46.0)	104 (60.1)
	No. missing	3	1
	Frontal lobe	219 (36.6)	74 (42.5)
	No. missing	2	0
	Parietal lobe	144 (24.0)	63 (36.2)
	No. missing	1	0
	Temporal lobe	248 (41.5)	98 (56.3)
	No. missing	3	0
	Occipital lobe	84 (14.0)	30 (17.2)
	No. missing	3	0
	Insula	77 (12.9)	52 (29.9)
	No. missing	3	0
	Corpus callosum	122 (20.4)	36 (20.7)
	No. missing	3	0
	Ventricle	163 (27.2)	101 (58.0)
	No. missing	2	0
	Midbrain	22 (3.7)	2 (1.1)
	No. missing	3	0
	Crossing midline	75 (12.7)	17 (9.8)
	No. missing	14	0
	Both hemispheres	65 (11.1)	19 (10.9)
	No. missing	14	0
	Right hemisphere	250 (42.6)	80 (46.0)
	No. missing	14	0
	Left hemisphere	272 (46.3)	75 (43.1)
	No. missing	14	0
**Intraoperative Add-Ons**	IONM	207 (35.4)	41 (23.7)
	No. missing	17	1
	Awake surgery	36 (8.0)	2 (1.2)
	No. missing	146	1
	Ultrasound	251 (41.8)	1 (0.6)
	No. missing	1	1
	5-ALA	230 (39.4)	88 (50.6)
	No. missing	17	0
	**Outcome measure**		
	No. missing	0	0
	GTR	259 (43.1)	112 (64.4)
	NRT	252 (41.9)	49 (28.2)
	STR	90 (15)	13 (7.5)

Features are organized into structured categories: Clinical Information, Tumor Topography, Tumor Location, and Intraoperative Add-Ons. Continuous variables are depicted as mean (±standard deviation). Categorical variables are shown as number of patients (%).

### External model validation

To evaluate the generalizability of the final prediction model, we validated it on an external cohort of 174 patients (Table 1 for details). Application of the model to this unseen dataset reaffirmed its robustness, yielding predictive performance comparable to the internal validation (Fig. 2A, B). The external validation resulted in an AUC of 0.78 (CI 0.73–0.82), confirming consistent classification performance across EOR categories (Fig. 2B). Discrimination performance for predicting GTR resulted in an AUC of 0.75 (CI 0.67–0.82), with precision and recall of 0.71 (CI 0.64–0.78) and 0.69 (CI 0.63–0.76), respectively. Predictions of NTR demonstrated an AUC of 0.59 (CI 0.50–0.69), a precision of 0.66 (CI 0.57–0.76), and a recall of 0.46 (CI 0.39–0.54). STR predictions resulted in an AUC of 0.69 (CI 0.53–0.85), a precision of 0.89 (CI 0.83–0.94), and a recall of 0.91 (CI 0.87–0.95). Calibration analysis using reliability plots and Brier scores (Supplementary Fig. 1B) revealed a moderate alignment between the predicted and observed probabilities. GTR predictions were overconfident, while NTR and STR tended to be underpredicted. Brier scores of 0.35 (GTR), 0.25 (NTR), and 0.52 (STR) reflected slightly reduced calibration compared to internal validation, supporting clinical applicability but also highlighting the need for recalibration in new institutional settings.

### Model examination

**Fig. 3:**
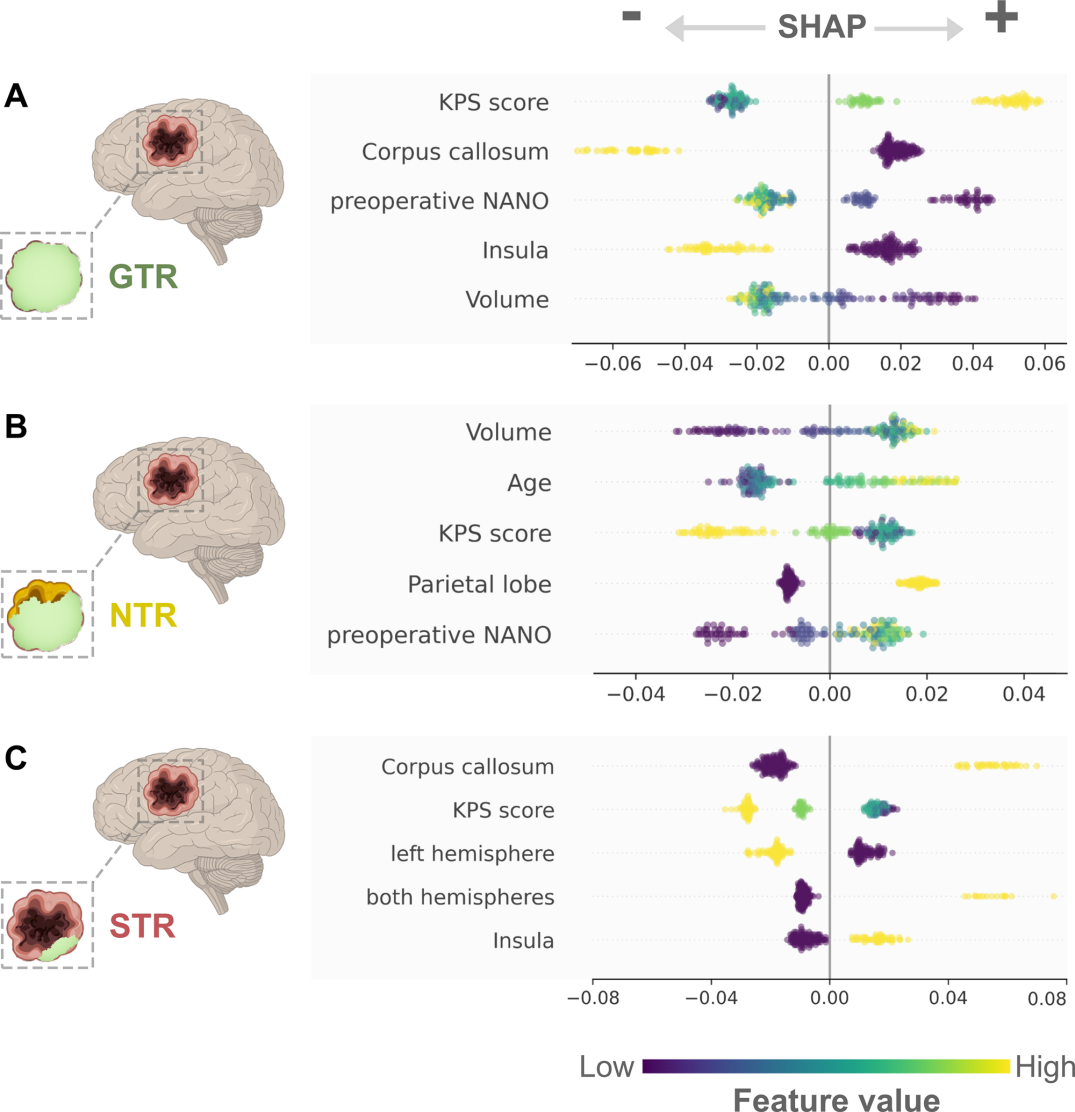
Explaining class predictions using SHAP. The SHAP approach provided an excellent tool for interpreting feature effects. The top five explanatory features were ranked in descending order for each class prediction based on their importance (cf. Supplementary Fig. 2). SHAP further allowed for interpreting each feature’s impact and direction on the prediction outcome. A positive SHAP value (x-axis) pushes the prediction towards the respective class outcome, e.g., predicting GTR. The feature value (viridis color scale) must also be considered to interpret each feature effect correctly. For predicting GTR (**A**), a higher KPS score (yellow) but a lower NANO score and tumor volume (both purple) pushed the model toward predicting GTR (positive SHAP value). For categorical features, e.g., the corpus callosum or insula, high values (yellow) indicate their infiltration and consequently lead to *not* predicting GTR (negative SHAP value). For the prediction of NTR (**B**), higher tumor volume, higher age, and a higher NANO score (all green/yellow) but a lower KPS score (purple) lead to the prediction of the class outcome (positive SHAP values). Tumor location in the parietal lobe (yellow) also led to the prediction of NTR (positive SHAP). Lastly, for STR (**C**), the infiltration of the corpus callosum and the insula or the involvement of both hemispheres (all yellow) positively impacted the prediction of STR (positive SHAP value). A higher functional status, indicated by a high KPS score (yellow) or the involvement of the left hemisphere, led to *not* predicting STR (negative SHAP value).

**Fig. 4:**
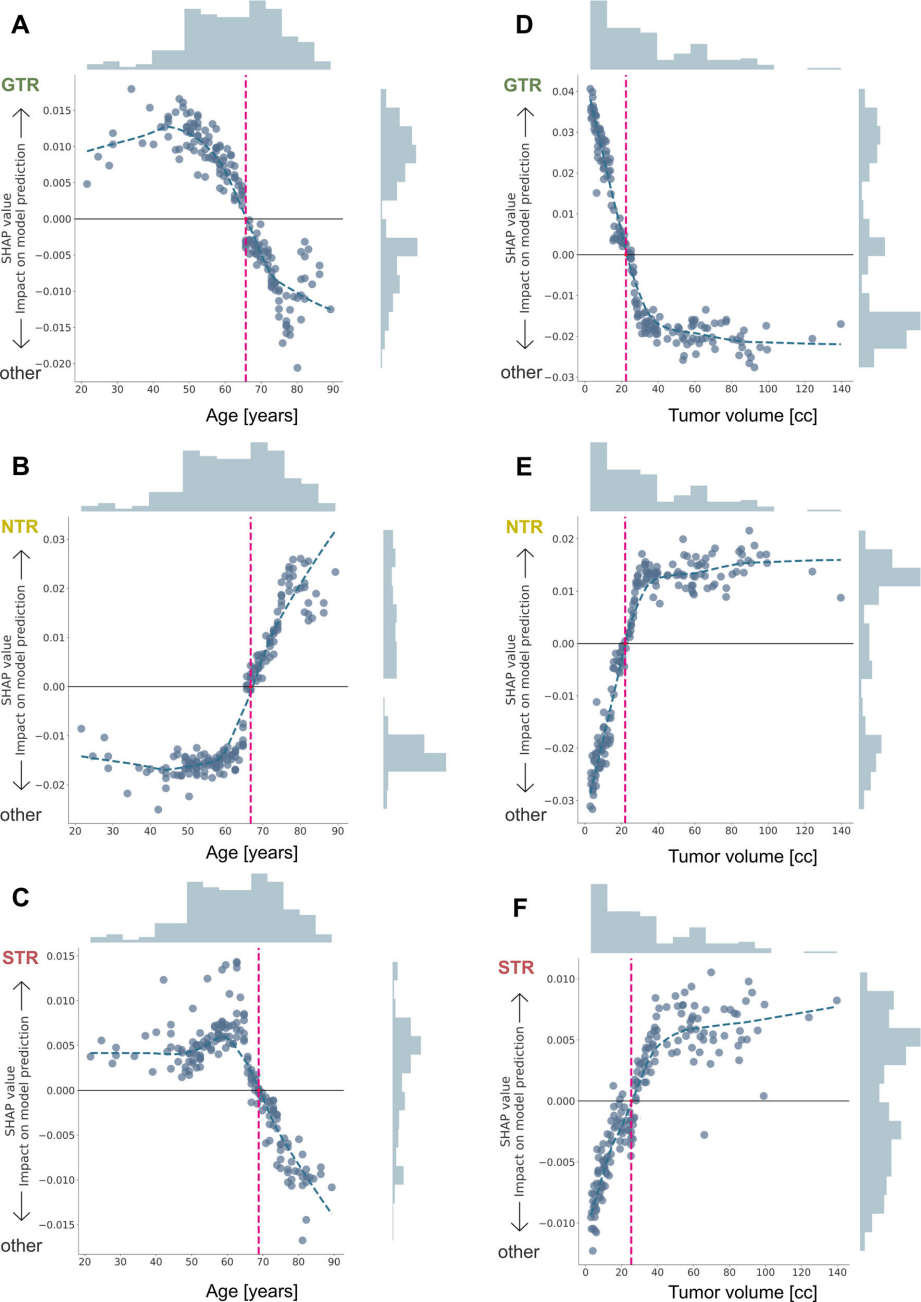
Inspecting age and tumor volume thresholds using SHAP. We used SHAP values (y-axes) to examine the continuous feature effects of *age* (blue) and *tumor volume* (green), which were among the most explanatory variables (cf. Supplementary Figs. 1–3**)**. A positive SHAP value steered the prediction towards GTR, NTR, or STR, whereas a negative SHAP value pushed the prediction towards *not* predicting that respective class outcome. Consequently, the intersection where y = 0 represents the age or tumor volume threshold (dotted pink line) at which the model’s prediction shifted between having a positive or negative impact on the class outcome. For patients’ age (**A**–**C**), the threshold fell between 66 to 69 years. We observed that for younger ages, the predictions of GTR (**A**) or STR (**C**) were more common, whereas as age increased, it exerted a stronger impact on the prediction of NTR (**B**). Regarding tumor volume (**D**–**F**), greater volume steered the predictions toward either NTR (**E**) or STR (**F**). The respective thresholds ranged from 22 to 25 cc. Tumor volumes with less than 22 cc were more inclined to shift the prediction towards GTR (**D**).

For predicting GTR, the five most explanatory features were the KPS score, the infiltration of the corpus callosum or insula, the preoperative NANO score, and tumor volume (Supplementary Fig 2A). We leveraged the SHAP framework to better understand the effect of each feature (see Supplementary Fig. 3–5 for all features). Preserved functional performance (Fig. 3A, high KPS in yellow), well-preserved neurological function (Fig. 3A, low NANO in purple), and small tumor volumes (Fig. 3A) strongly favored GTR predictions. A tumor volume below 22 cc shifted predictions toward GTR (Fig. 4A), while infiltration of the corpus callosum or insula reduced the model’s confidence in achieving complete resection.

The most relevant features for predicting NTR included tumor volume, patient age, parietal lobe involvement, KPS, and the NANO score (Supplementary Fig 2B). Lower functional performance (low KPS) and severe neurological impairment (high NANO) increased the probability of NTR (Fig. 3B), as did larger tumor volumes, particularly in the parietal lobe, and older patient age. Moreover, we inspected the respective age and volume thresholds for predicting NTR (Fig. 4B, E). We observed that for younger ages, the likelihood of predicting GTR (<66 years**)** or STR (<67 years**)** was notably higher (Fig. 4A, C). Conversely, with advanced age (>69 years, Fig. 4B), the influence on predicting NTR became more pronounced.

Predictions for STR increased with the infiltration of the corpus callosum and insula, bihemispheric involvement (Fig. 3C, all in yellow), and lower functional status (Fig. 3C, KPS, depicted in purple). The isolated involvement of the left hemisphere led to not predicting STR. Tumor volumes exceeding 25 cc further reinforced STR predictions (Fig. 4F), particularly in patients under 69 years of age. In older patients, model confidence shifted toward predicting NTR (Fig. 4B, C).

The model reflects real-world considerations of surgical risk associated with tumor location, functional reserve, age, and volume, which neurosurgeons routinely integrate when estimating resectability^18^. The alignment between model behavior and expert practice might indicate that the model has effectively internalized the multifactorial decision-making framework that underlies expert surgical judgment.

### Sensitivity analysis

**Fig. 5:**
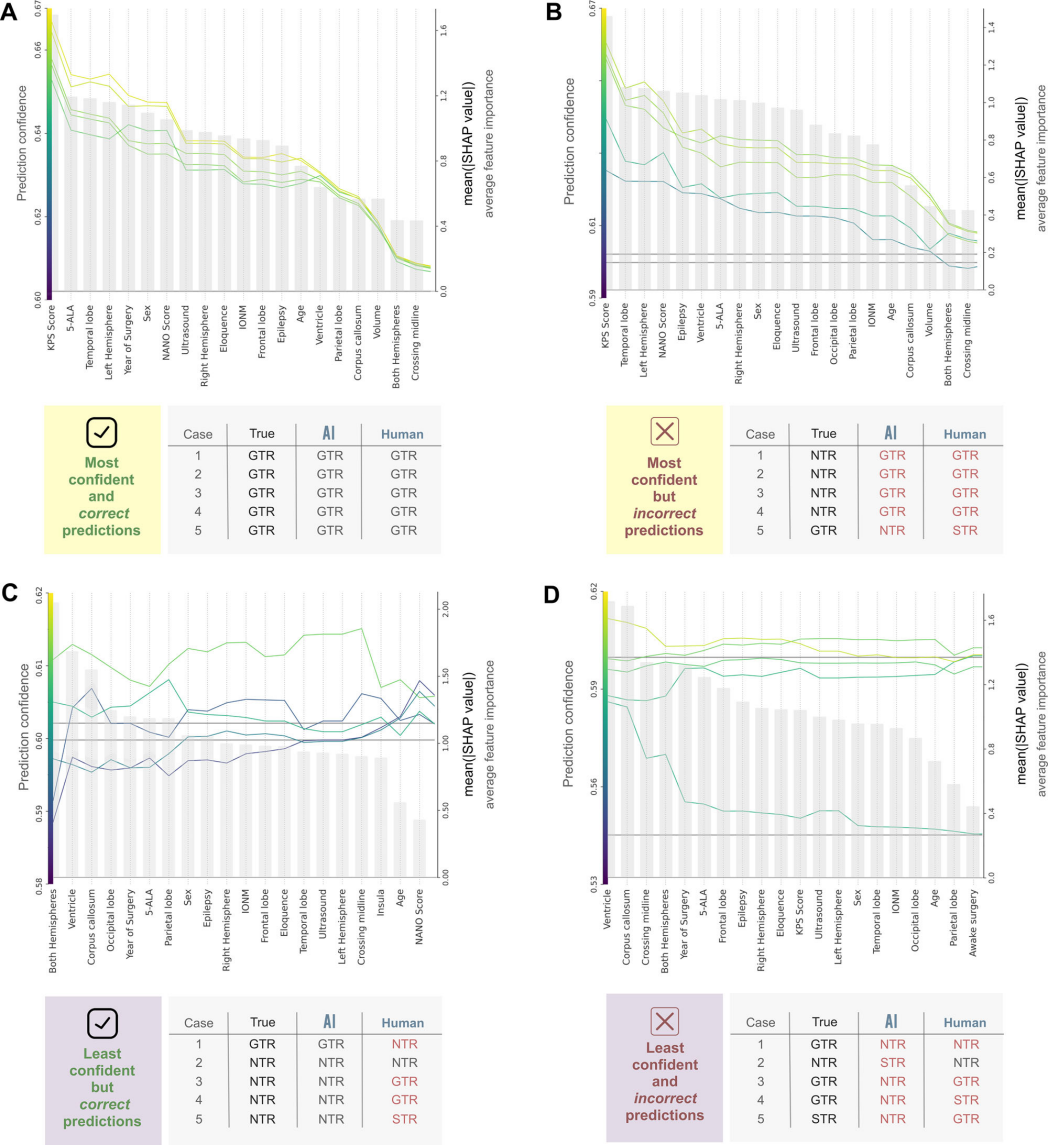
Sensitivity analysis. To better comprehend the strengths and limitations of our AI model, we performed a sensitivity analysis^19^. For that purpose, we closely inspected the top five cases in which our classification model was most confident and correct (**A**), most confident and incorrect (**B**), least confident and correct (**C**), and least confident and incorrect (**D**). For each examination, we compared the performance of the AI algorithm against the performance of a human expert rater. (**A**) First, the top five instances in which our AI model exhibited the highest confidence and correctness corresponded to all GTR cases and aligned well with the human-rated assessment. Features were ranked in order of their absolute importance (gray bar plot, represented on the right y-axis by their mean(|SHAP value|)). Notably, the most substantial shifts in explanatory value, characterized by sharp increases, were consistently associated with tumor volume, NANO, and KPS scores, corroborating our prior results (cf. Fig. 2). Inspecting the most confident yet incorrect AI predictions (**B**), we noticed equally incorrect predictions by the human rater. Most instances that were incorrectly estimated were NTR cases. The substantial increases in the SHAP curves were again linked to functional performance (KPS score) and neurological impairment (NANO score), tumor volume, and infiltration of the corpus callosum. These findings closely mirrored our previous NTR SHAP explanations displayed in Fig. 3B. Similarly, most instances that our AI model predicted correctly, albeit with low confidence, resembled true NTRs (**C**). However, the bar plots for feature importance highlighted that in these low-confidence cases, the model’s decision did not stem from a single dominant feature but from the aggregate effect of multiple mid-strength contributors (e.g., both hemispheres, ventricle, corpus callosum). Their combined influence crossed the probability threshold, explaining why small changes affected the classification. A high degree of intersubject variability became evident after a closer examination of the corresponding inconsistent SHAP curves. For the same feature, e.g., eloquence in cases 1 and 4, some cases negatively influenced the prediction (characterized by a curve decrease), while others displayed a positive impact (indicated by increasing curves). Notably, these specific cases pose comparable challenges for the human rater, with a majority (4 out of 5) being incorrectly predicted. In such instances, the AI tool may enhance surgical decision-making by offering valuable and explainable insights. Finally, when considering the least confident and incorrect AI predictions, we observed a comparable difficulty level for the human rater (with 4 out of 5 incorrect estimations). The SHAP curves in these cases displayed shallower changes, indicating less substantial influences from individual features. The less pronounced curves underscored the complexity of those predictions, relying on many subtle feature interactions, and highlighted the areas where both human expertise and AI capabilities encounter similar challenges. Gray horizontal lines in Panels B–D mark class-specific decision thresholds on the model output, helping to visualize how feature contributions shift predictions relative to the model’s baseline output (gray line). Their number and placement vary according to the prediction values. For example, no lines appear in Panel A, as all predictions exceed the threshold and lie outside the y-axis range.

To understand the strengths and limitations of the model, we conducted a sensitivity analysis^19^ by assessing four types of prediction results: most *confident and correct, most confident and incorrect, least confident and correct*, and *least confident and incorrect* (Fig. 5). We compared the AI’s prediction with a human expert rater in each case:

In the *most confident and correct* predictions, all top five cases corresponded to GTR (Fig. 5A). Both AI and human expert predictions aligned well. We ranked the corresponding features in the prediction according to their absolute importance (Fig. 5, right y-axis), with the most significant shifts in explanatory value for tumor volume, NANO, and KPS scores, consistent with earlier results (cf. Fig. 3).

In the *most confident but incorrect* predictions (Fig. 5B), the model’s errors mirrored those of the human rater, with the majority of misclassified cases falling into the NTR class. Marked shifts in SHAP value curves, particularly involving KPS, NANO, and tumor volume, echoed patterns seen in earlier NTR predictions (Fig. 3B). These large positive contributions reflecting patients’ functional status may override negative signals from other features, e.g., tumor volume (see Supplementary Fig. 11 on failure modes in high-confidence prediction errors). These high-confidence errors reveal that, when favorable functional status and low NANO scores align, the model can over-weight other contradicting inputs and effectively “drown out” the counter-signal from volume effects, leading to confidently erroneous GTR predictions.

In the *least confident yet correct* predictions (Fig. 5C), most were also NTR cases. These examples showed larger intersubject variability in SHAP curves, with the same feature (e.g., eloquence) influencing predictions differently across cases. The complex variability mirrored the challenges faced by the human rater, who misclassified 4 out of 5 of these cases. In contrast to the confident cases, no single feature dominated in low-confident correct cases. Instead, the model balanced several mid-strength contributing feature effects just above the respective probability threshold, making it sensitive to small perturbations in feature values.

In the *least confident and incorrect* predictions, a comparable difficulty level was observed for both the AI and human rater (Fig. 5D). The SHAP curves in these patient predictions displayed slight changes, signifying less notable influences of single features. Such flat patterns suggest that these were inherently complex cases with no dominant predictive signals, but rather many subtle feature interactions, highlighting limitations shared by both human expertise and AI capabilities.

### Clinical impact evaluation

To evaluate the impact and added value of human-AI collaboration in clinical decision-making, we prospectively assessed ten cases by comparing the performance of a human expert (the gold standard), the standalone AI model, and a combined human-AI approach. The collaborative rater had access to the AI model’s local predictions and visual explanations (see Supplementary Fig. 6 for an exemplary model output).

The standalone AI model showed a robust performance similar to the external validation results, with an average accuracy of 0.73 (see Table 2 for overall performance metrics, see Supplementary Fig. 10 for confusion matrices), and class-specific accuracies (see Table 3 for class-specific performance metrics) for GTR and NTR of 0.67 (0.40–0.90) and for STR of 0.78 (0.50–1.00). Whereas the human rater showed a lower overall accuracy of 0.53 with class-specific accuracies for GTR 0.44 (0.10–0.80), NTR 0.33 (0.10–0.70), and higher accuracy for STR 0.89 (0.70–1.00). However, the collaboration between the human expert and the AI model notably improved performance, achieving an overall accuracy of 0.94 across all averaged EOR classes, with class-specific accuracies of 0.89 (0.70–1.00) for GTR and NTR, and 1.00 (1.00–1.00) for STR. The use of the AI application advanced the expert estimations, improving precision and recall. The F1 score provides a single metric that balances precision and recall. Here, we observed an improvement of F1 scores of ∆ 0.44, ∆ 0.57, and ∆ 0.14 for GTR, NTR, and STR, respectively. Averaged across EOR classes, the overall F1 score improved from 0.3 to 0.92. The level of agreement between the ground truth and the estimations generated through the human-AI collaboration was higher (Cohen’s κ = 0.84) than the agreement observed between the ground truth and the human-generated estimations alone (Cohen’s κ = 0.41). These findings demonstrate that integrating AI support can meaningfully enhance expert judgment and promote more accurate and nuanced clinical predictions.

**Table 2:** Overall performance metrics for the human rater, AI, and the Human-AI collaboration

	Human	AI	Human-AI
**Precision**	0.30 [0.00–0.56]	0.41 [0.15–0.53]	0.94 [0.86–1.00]
**Recall**	0.34 [0.08–0.58]	0.50 [0.33–0.67]	0.92 [0.75–1.00]
**F1-Score**	0.30 [0.07–0.52]	0.42 [0.21–0.59]	0.92 [0.71–1.00]
**Accuracy**	0.54 [0.40–0.67]	0.73 [0.60–0.87]	0.94 [0.80–1.00]

Reported values are bootstrap means with 95% confidence intervals (stratified bootstrap, *n* = 1000). Overall accuracy (proportion of correctly predicted cases), precision, recall, and F1-scores are summarized as macro-averages across all classes.

*GTR* gross-total resection, *NTR* near-total resection, *STR* subtotal resection.

**Table 3:** Comparing class-specific performance metrics for the human rater, AI, and the Human-AI collaboration

		Human	AI	Human-AI
**Accuracy**	GTR	0.44 [0.10 - 0.80]	0.67 [0.40 - 0.90]	0.89 [0.70 - 1.00]
	NTR	0.33 [0.10 - 0.70]	0.67 [0.40 - 0.90]	0.89 [0.70 - 1.00]
	STR	0.89 [0.70 - 1.00]	0.78 [0.50 - 1.00]	1.00 [1.00 - 1.00]
**Precision**	GTR	0.50 [0.12 - 0.87]	0.81 [0.25 - 0.93]	0.92 [0.81 - 1.00]
	NTR	0.36 [0.06 - 0.79]	0.68 [0.29 - 1.00]	0.94 [0.85 - 1.00]
	STR	0.87 [0.49 - 1.00]	0.62 [0.25 - 1.00]	1.00 [1.00 - 1.00]
**Recall**	GTR	0.44 [0.10 - 0.80]	0.67 [0.40 - 0.90]	0.89 [0.70 - 1.00]
	NTR	0.33 [0.10 - 0.70]	0.67 [0.40 - 0.90]	0.89 [0.70 - 1.00]
	STR	0.89 [0.70 - 1.00]	0.78 [0.50 - 1.00]	1.00 [1.00 - 1.00]
**F1-Score**	GTR	0.44 [0.13 - 0.80]	0.64 [0.33 - 0.90]	0.88 [0.64 - 1.00]
	NTR	0.33 [0.07 - 0.68]	0.65 [0.29 - 0.91]	0.90 [0.69 - 1.00]
	STR	0.86 [0.58 - 1.00]	0.69 [0.33 - 1.00]	1.00 [1.00 - 1.00]

Metrics are shown for each class, which are GTR, NTR, and STR (95% confidence intervals).

*GTR* gross-total resection, *NTR* near-total resection, *STR* subtotal resection.

## Discussion

We developed and externally validated an explainable AI model capable of predicting EOR in GBM patients. By leveraging machine learning and the SHAP framework, our prognostic model offers interpretable, individualized EOR risk assessments, effectively addressing the challenge of predicting and understanding each patient’s tumor resectability. Most importantly, when combined with expert judgement, the *human-AI collaboration* outperformed both expert clinicians or AI alone, underscoring the synergistic potential of human intuition and the predictive capabilities of AI. By enabling precise preoperative EOR predictions, our approach may successfully contribute to tailored patient care by advancing personalized decision-making in neuro-oncology.

Surgical resection has the most impact on overall survival in GBM by removing both the tumor mass and areas of tumor invasion. A comprehensive meta-analysis confirmed that greater EOR is associated with improved overall and progression-free survival^7^. Other studies have shown a significant decrease in the relative death rate for GBM patients undergoing resections that exceed 80% of CE-tumor removal^20,21^, suggesting a “dose-dependent” effect between EOR and survival, beginning at this threshold. A recent study further refined this understanding by identifying two distinct epigenetic GBM subtypes (high and low neural), each differing in their peritumoral connectedness, clinical course, and prognosis^10^. Notably, the study suggested that these GBM subtypes benefit differently from the EOR, coining the term “molecular-guided resection” for the first time and highlighting the need for patient-tailored resection strategies. At the same time, surgical planning must carefully navigate the delicate balance between achieving maximal resection and avoiding neurological harm, as the advantages of an extensive resection may reduce or vanish entirely with new neurological deficits^22^. Conversely, leaving any CE tissue unremoved resulted in clinical trajectories similar to those of patients who received no resection at all^8,23^.

The increasing complexity of surgical decision-making, driven by emerging molecular markers and the importance of maximizing EOR while preserving neurological function, underscores the need for a reliable and reproducible tool to estimate EOR preoperatively. To ensure clinical safety and transparency, we adhered to the established AI reporting standards^19,24–26^ and validated our model both internally and externally. It demonstrated generalizability and consistent performance across EOR classes, a crucial aspect for its clinical applicability. Contrary to the common misconception that model complexity enhances accuracy^16,27,28^, our findings support that this is not necessarily the case, especially in structured data with inherently meaningful features. Although deep learning models excel in areas including image, speech, or language processing, tree-based models have been consistently more effective on tabular-style datasets^17,29,30^. Highlighting the strengths of tree-based models^17,29,31^, such inherently explainable models can be more accurate than deep learning techniques in many clinical applications^32–34^.

The value of our approach lies not just in its predictive accuracy but also in its explainability, which may enhance trust and support more transparent clinical decision-making. A key strength of the approach was identifying critical features and their hidden interactions that could otherwise remain obscured to the human rater. Typically, human predictions and prior rating systems for EOR relied mainly on isolated tumor features, such as anatomical location and volume^13,35^. In contrast, our model frequently highlighted patient-specific clinical features, such as KPS and NANO scores, as primary drivers of EOR predictions, therefore, redirecting attention toward features that might otherwise be underweighted in surgical judgment. Explainable AI approaches further add value by contextualizing the effects of individual features within the context of others^16,27,36^, a capability often overlooked by human raters who tend to assess features in isolation. The complexity of interpatient variability became particularly relevant in NTR cases, where our model complemented human judgment by highlighting individual features, thereby improving predictive accuracy when combined with human insights. In addition, the human-AI collaboration achieved near-perfect performance in STR prediction; however, given the preliminary nature of this pilot evaluation, the possibility of overfitting must be warranted. At the same time, this strong performance appears clinically plausible, as surgical failure may be easier to predict than success: STR typically reflects clear-cut scenarios such as tumor infiltration of highly eloquent regions or large residual volumes. In contrast, the distinction between NTR and GTR may often lie in subtle differences, explaining why predictive performance was lower in this more ambiguous ‘gray zone.’

Lastly, our findings illustrated that a synergistic human-AI collaboration outperformed both the physician expert and the standalone machine learning model in a proof-of-concept, early-stage impact evaluation, demonstrating the advantage of combining AI with human expertise. We demonstrated improved performance of the human-AI approach compared to the current gold standard, the neurosurgical expert opinion alone. However, our pilot evaluation was limited in size and scope and must be confirmed in larger, prospective studies integrating long-term survival and functional outcome data to fully characterize its clinical utility and impact. This synergistic framework aligns with the broader vision for AI in medicine^37,38^: not to replace human expertise, but to augment it as a decision-support tool that enhances clinical reasoning. Our results are consistent with the existing literature, which suggests that the accuracy of predictive AI systems, although notable, does not warrant their standalone deployment in clinical settings. Instead, hybrid approaches that combine human expertise with AI capabilities have shown greater promise across various clinical applications^39–41^. In our study, the combined approach improved prediction performance and supported more informed and nuanced clinical decision-making. Therefore, such an effective partnership between clinicians and AI, as demonstrated in our study, could redefine preoperative planning and patient counseling within neuro-oncology.

However, several limitations should be acknowledged. Although SHAP improves model transparency by visualizing feature contributions^29,42^, it remains a post-hoc method that does not infer causality and may produce ambiguous interpretations in the presence of nonlinear interactions. While SMOTE helped to address class imbalance during training, the generation of synthetic samples, particularly for the minority NTR class, may not fully capture real-world variability and could limit clinical applicability. The underrepresentation of NTR cases in the external validation cohort may have further reduced discriminative performance. Despite precautions during training and the use of an independent external validation cohort, some degree of overfitting cannot be fully excluded, underscoring the need for larger and more balanced multicenter studies. The decline in calibration performance likely also reflects the inherent heterogeneity of real-world data. Features with substantial missingness also warrant cautious interpretation. Despite using a structured imputation approach, residual bias from missing data may affect model reliability. The external validation cohort originated from a single center and had fewer NTR cases, which may have contributed to the model’s reduced generalization and lower discriminative performance for that class. Additionally, variability in institutional philosophies and individual surgeon preferences, while being partly mitigated by our multicenter design, was not explicitly modeled and may have introduced further heterogeneity. However, our interpretability analyses using SHAP confirmed that center-specific surgical adjuncts (e.g., intraoperative ultrasound, IONM) contributed minimally to predictions, indicating that the model’s performance was not substantially influenced by local practice variations. While the model performed best in predicting GTR, its reduced accuracy in NTR cases, which represent clinically ambiguous and borderline cases that often depend on individual surgical skill and experience, warrants consideration of its practical implications for surgical decision-making. Further advancements of this approach could incorporate automated extraction of imaging features, such as radiomic and deep learning-derived biomarkers^43–46^, or even intraoperatively obtained molecular profiles to support more objective and molecular-based resection planning. Additionally, the identified failure modes highlight the need to explore explicit interaction terms to better capture complex feature relationships. Together, these findings underscore the importance of further larger prospective evaluation, carefully fine-tuning and adapting models in new clinical settings, and reinforce the need for human oversight, particularly when algorithmic confidence is low.

Our study highlighted the role of predicting EOR in optimizing surgical outcomes for GBM patients. By developing an explainable model that enhanced the preoperative estimation of EOR, we provided a valuable add-on decision support tool for personalized treatment planning. However, our findings also highlight the challenges of translating predictive AI into real-world clinical practice. By embedding interpretability into our approach, we enable clinicians to understand the model’s predictions, thereby promoting informed decision-making rather than blind reliance on algorithmic outputs. Finally, the value of the human-AI collaboration underscores the model’s real-world utility. Integrating transparent AI systems into GBM management may help reduce variability in clinical judgment, enhance precision in surgical strategy, and ultimately advance personalized care in neuro-oncology.

## Methods

To maximize clinical safety and enable a direct and transparent assessment of the clinical impact of the proposed AI application, we designed the workflow shown in Fig. 1 in accordance with established AI reporting guidelines^19,24–26^. In detail, four key components of our approach are highlighted: (**A**) multi-center data collection to capture real-world variability, (**B**) methodological rigorous model development and validation, (**C**) a multi-layered interpretability framework including global and local SHAP explanations, the assessment of individual predictions, and multiple sensitivity analyses, and lastly (**D**) the comparative evaluation against expert estimations.

### Patient population

We analyzed a multicenter registry with 811 patients diagnosed with de novo GBM (WHO grade 4, IDH-wildtype) who underwent microsurgical resections. Every site pursued a maximal-safe-resection philosophy^47,48^, that is, maximizing tumor removal while preserving neurological function, thereby unifying surgical approaches across all participating institutions. However, individual surgeon preferences were not explicitly modeled. The inclusion criteria of this study were: (1) adult newly-diagnosed GBM (age above 18 years), (2) all patients received primary intracranial tumor surgery, (3) diagnostic biopsies and recurrent cases were excluded, (4) availability of pre- and early postoperative MRI imaging, and the primary outcome (EOR). Data for model development and internal validation were retrospectively collected at five international centers (Table 1, see Supplementary Table 6 for center-stratified characteristics): Aachen, Hamburg, and Cologne (Germany), Denver (USA), and Zurich (Switzerland). Data from a sixth institution (Bielefeld, Germany) was reserved for external validation. To demonstrate the clinical benefit of our approach, we prospectively tested the validated framework in ten consecutively recruited patients (see Supplementary Table 2 for cohort details) with suspected de novo WHO IV GBM (subsequently confirmed on histopathology), applying the same eligibility criteria as in model development and internal validation (adult; primary intracranial resection; availability of pre- and early postoperative MRI to define EOR). The study protocol was approved by the local ethics committee review board (EK 142-20). All research was conducted in accordance with the Declaration of Helsinki. Patients provided informed consent, or the local IRB waived consent requirements based on the study’s retrospective nature and minimal risks to participants.

### Characteristics of the glioblastoma patient cohort

Demographic, clinical, and imaging features were recorded for each patient and served as input variables for model development. Available sociodemographic and clinical features included age at surgery, year of surgery, sex, the incidence of preoperative epilepsy, preoperative Karnofsky Performance Status (KPS), and the NANO score (Neurologic Assessment in Neuro-Oncology), which provided an assessment of objective clinician-reported neurologic function^49^. Specifically aimed to evaluate neurologic function among brain tumor patients, the NANO score covers nine relevant domains frequently impacted by brainstem, supra-, and infratentorial lesions. The nine key domains include the rating of gait, strength, ataxia, sensation, visual fields, language, level of consciousness, and behavior. Scoring ranges from 0 (no neurological deficit) to a maximum of 23 (most severe deficits across all domains). Tumor-related features were assessed by experienced local neurooncological and neuroradiological attendings, including the anatomical location (frontal, temporal, parietal, occipital lobe, the infiltration of the insula or the midbrain) based on standardized, well-defined anatomical parcellations^50,51^, the proximity to the ventricle, the incidence of crossed midlines, hemispheric lateralization, and volumetric tumor size (in cc, using well-established 3D-lesion segmentation software, e.g., Brainlab) of the contrast-enhancing tumor component. Presumed eloquent brain areas included the sensorimotor strip (precentral and postcentral gyri), dominant hemispheric perisylvian language areas (superior temporal, inferior frontal, and inferior parietal areas), basal ganglia, thalamus, and visual cortex^52^.

### Outcome measures characterized by detailed multiclass representations

The individual EOR was the primary endpoint of this machine-learning investigation. EOR was assessed according to a standardized imaging protocol reflecting the current gold-standard approach to postoperative glioblastoma evaluation and in line with previously established assessment criteria^53–55^. Following both protocols, experienced neurosurgeons evaluated multisequence postoperative contrast-enhanced (CE) MRI scans obtained within 72 hours of surgery alongside the neuroradiology report. The optimal threshold for EOR needed to improve patient outcomes remains a topic of ongoing debate and lacks consensus among experts^9,56,57^. Most studies have focused on the binary differentiation between GTR and STR^58^. However, even a small residual tumor would be classified as STR, which may lead to a potential blurring of the critical distinction between an NTR, e.g., a small leftover CE rim with overall reasonable cytoreduction, and a substantial subtotal resection. The absence of a clear distinction between NTR and STR can impose limitations on interpreting further conclusions. Therefore, we used a *multiclass framework of EOR* to appreciate even slight subtleties^53^. For this study, EOR was divided into three distinct classes: GTR (0% of CE tumor left), NTR (<10% of CE tumor left), and STR (>10% of CE tumor left).

### Data preprocessing and resampling

To ensure reliable model learning, feature engineering was used to standardize and structure the heterogeneous input data. Categorical variables with more than two categories were one-hot encoded, and continuous features were adjusted to a comparable scale by mean centering to zero and variance scaling to one. Missing values were imputed using a k-nearest neighbor approach co-trained during model development and applied to unseen new data^59^. This imputation strategy estimates missing values based on feature similarity across the dataset, preserving the multivariate structure of the data (see Supplementary Figs. 12 and 13 for sensitivity analyses supporting the choice of k). To assess and mitigate potential bias, we conducted sensitivity analyses for features with higher missingness (>20%) to assess whether the inclusion and imputation of these features meaningfully distorted model performance (Supplementary Fig. 7-9). The Synthetic Minority Over-sampling Technique (SMOTE) was applied to mitigate class imbalance^60^.

The development dataset was randomly split into a training/testing set and a held-out internal validation set (80:20, stratified for the outcome) for model development and internal validation, respectively. Only the final machine learning model was brought forward to the external cohort, providing the highest level of validation^19,61^.

### Machine learning model development and evaluation

A set of supervised classifiers optimized to the multiclass objective was assessed during model selection. The learning algorithms embraced different model representations, including instance learners (Support vector classifier, Nu-support vector classifier), hyperplane learners (logistic regression), Gaussian Naive Bayes, a low-dimensional neural network, and tree-based algorithms with increasing model complexity (Decision Trees, random forest, AdaBoost, Gradient Boosting, and Explainable Boosting Machines)^31,62,63^. An analytical emphasis was placed on *limited model complexity* and *increased interpretability*, given the tabular nature of the data and the aim to promote accessible clinical application and interpretation.

The most competitive model was selected in a data-driven fashion based on the multiclass one-versus-rest metric area under the curve (AUC) in fivefold nested cross-validation (stratified on the outcome variable) on the training set. Hyperparameter tuning was performed using a grid-search approach. For the random forest classifier, we systematically varied the number of estimators, tree depth, and the minimum number of samples required to split a node or form a leaf. These parameters inherently regularize model complexity and promote stable embedded feature selection by prioritizing only the most predictive variables^17,64^. This embedded feature selection mechanism is particularly suited for multi-class settings^64,65^, where individual features may contribute differentially across classes. The model parameters were fit in 1,000 bootstrap iterations to estimate 95% confidence intervals. Calibration was visually assessed using calibration curves and quantitatively evaluated using Brier scores (0–1; a score of 0 indicated perfect model calibration, and a score of 1 corresponded to poor model calibration). Discrimination and calibration performance metrics are reported separately for the internal and external validation cohorts. To measure the level of agreement between the ground truth, the AI, and human-AI collaboration, we calculated the Cohen κ coefficient^66,67^. Similar to correlation coefficients, Cohen’s κ can range from -1 to +1, with 0 indicating random chance and 1 signifying a perfect agreement among raters. Generally, performances with κ between 0.2 and 0.4 can be interpreted as fair, and κ > 0.8 as substantial^66,68^.

### Model interpretation using Shapley Additive Explanations (SHAP)

Understanding *how* complex models make specific predictions is as critical as their predictive performance^36^. To better understand how ML models infer their predictions, we applied the game-theory-inspired SHapley Additive exPlanations (SHAP) framework^29,42^. This unified approach enhances interpretability by computing Shapley values to estimate feature importance for otherwise considered ‘black-box’ models. In contrast to other explanation methods, SHAP provided at least three significant advantages for the goal of the current study: (1) SHAP enables the optimal computation of *local explanations* with theoretical guarantees of consistency, (2) it captures *local interaction effects* between features, extending beyond simple additive models. (3) SHAP aggregates many local explanations to represent *global structure* while retaining local faithfulness to the original model. This dual capability allows SHAP to interpret both local explanations for *individual predictions* (local—for an individual patient, cf. Supplementary Fig. 6) and *overall model behavior* (*global*—across patients or classes, cf. Fig. 3). It extends traditional feature importance methods, such as absolute impact or ranking approaches, by quantifying not only the magnitude of a feature’s impact but also its prevalence and direction.

## Supplementary information

Supplementary Information

## Data Availability

Data are available upon reasonable request.

## Code availability

The complete source code is openly accessible at http://github.com/jkernbach/xAI_glioblastoma.
