# Supplementary material for: Enhancing decision-making in glioblastoma surgery through an explainable human-AI collaboration: an international multicenter model development and external validation study
Source: NPJ Precis Oncol. 2025 Nov 27;9:387. doi: 10.1038/s41698-025-01183-2 (PMC12672623; doi:10.1038/s41698-025-01183-2)
Supplement: Supplementary file 1 — Supplementary Information [file 41698_2025_1183_MOESM1_ESM.pdf]

Supplementary Materials

Classifier	Model performance AUC (micro)	AUC (macro)
Random forest	0.83	0.81
Gradient Boosting	0.81	0.79
SVC	0.81	0.80
NuSVC	0.81	0.79
EBM	0.80	0.79
AdaBoost	0.77	0.75
Logistic regression	0.71	0.71
Decision tree	0.70	0.70
Gaussian Naïve Bayes	0.69	0.70
MLP	0.56	0.55

Supplementary Table 1. Model selection

Within the model selection, different learning algorithms were assessed. All classifiers embraced different model representations, including instance learners (Support vector classifier (SVC), Nu-support vector classifier (NuSVC)), hyperplane learners (logistic regression), Gaussian Naive Bayes, a low-dimensional neural network (MLP), and tree-based algorithms with increasing model complexity (Decision Trees, random forest, AdaBoost, Gradient Boosting, and Explainable Boosting Machines (EBM)) [34–36]. The most competitive model, the random forest, was selected based on the multiclass one-versus-rest metric *area under the curve* (AUC) in fivefold nested cross-validation (stratified on the outcome variable) on the training set during model development.

Feature	Clinical impact cohort (n=10)
Female sex	4 (40)
Age	66.8 ± 6.1
Tumor volume	35.8 ± 26
Preoperative NANO score	2.3 ± 2.1
Preoperative KPS score	78 ± 13.2
Epilepsy	2 (20)
Eloquence	7 (70)
Frontal lobe	2 (20)
Parietal lobe	4 (40)
Temporal lobe	7 (70)
Occipital lobe	1 (10)
Insula	1 (10)
Corpus callosum	1 (10)
Ventricle	3 (30)
Midbrain	1 (10)
Crossing midline	0
Both hemispheres	0
Right hemisphere	2 (20)
Left hemisphere	8 (80)
IONM	7 (70)
Awake surgery	1 (10)
Ultrasound	0
5-ALA	7 (70)
**Outcome measure**	
GTR	4 (40)
NTR	4 (40
STR	2 (20)

Supplementary Table 2. Prospective cohort to test for clinical impact.

Patients were suspected de novo WHO IV glioma at enrollment and later histologically confirmed, and the same inclusion criteria as the development/validation cohorts were applied (adult GBM; primary intracranial resection; pre- and early postoperative MRI available for EOR). Continuous variables are depicted as mean (± standard deviation). Categorical variables are shown as the number of patients (%).

		GTR<br>n = 259	NTR<br>n = 252	STR<br>n = 90
**Clinical Information**	Age (years)	62 (55, 70)	66 (56, 73)	63 (56, 69)
	Female	102 (39%)	97 (38%)	38 (42%)
	KPS	85 (80, 100)	80 (70, 90)	80 (60, 80)
	NANO	1 (0, 3)	2 (1, 3)	2 (1, 4)
	Epilepsy	79 (31%)	63 (25%)	21 (24%)
**Topography**	Eloquence	99 (38%)	129 (52%)	47 (53%)
	Volume	45 (84)	52 (83)	50 (84)
	Frontal Lobe	95 (37%)	91 (36%)	33 (37%)
	Parietal Lobe	44 (17%)	79 (31%)	21 (23%)
	Temporal Lobe	117 (45%)	101 (40%)	30 (34%)
	Occipital Lobe	34 (13%)	44 (18%)	6 (6.7%)
	Insula	19 (7.3%)	42 (17%)	16 (18%)
	Corpus Callosum	32 (12%)	54 (22%)	36 (40%)
	Ventricle	49 (19%)	84 (34%)	30 (33%)
	Midbrain	6 (2.3%)	5 (2.0%)	11 (12%)
	Crossing Midlines	17 (6.8%)	36 (15%)	22 (25%)
	Both Hemispheres	12 (4.8%)	27 (11%)	26 (29%)
	Right Hemisphere	107 (42%)	112 (46%)	31 (35%)
	Left Hemisphere	133 (53%)	107 (43%)	32 (36%)
**Intraoperative Add-Ons**	5-ALA	113 (46%)	81 (33%)	36 (41%)
	IONM	91 (37%)	86 (35%)	30 (34%)
	Awake Surgery	15 (7.8%)	12 (5.7%)	9 (18%)
	Ultrasound	92 (36%)	106 (42%)	53 (59%)

Supplementary Table 3. Characteristics of the Development Cohort stratified by EOR.

Continuous variables are presented as mean ± standard deviation or median (interquartile range), as appropriate; categorical variables are reported as count (percentage).

Study design (Part 1)	Completed: page number		Notes if not completed
The clinical problem in which the model will be employed is clearly detailed in the paper.	☒	3	
The research question is clearly stated.	☒	3-4	
The characteristics of the cohorts (training and test sets) are detailed in the text.	☒	5	
The cohorts (training and test sets) are shown to be representative of real-world clinical settings.	☒	5	
The state-of-the-art solution used as a baseline for comparison has been identified and detailed.	☒	7	
**Data and optimization (Parts 2, 3)**	**Completed: page number**		**Notes if not completed**
The origin of the data is described and the original format is detailed in the paper.	☒	12	
Transformations of the data before it is applied to the proposed model are described.	☒	13-14	
The independence between training and test sets has been proven in the paper.	☒	14	
Details on the models that were evaluated and the code developed to select the best model are provided.	☒	14	
Is the input data type structured or unstructured?	☒ Structured ☐ Unstructured		
**Model performance (Part 4)**	**Completed: page number**		**Notes if not completed**
The primary metric selected to evaluate algorithm performance (e.g., AUC, F-score, etc.), including the justification for selection, has been clearly stated.	☒	14	
The primary metric selected to evaluate the clinical utility of the model (e.g., PPV, NNT, etc.), including the justification for selection, has been clearly stated.	☒	13-14	
The performance comparison between baseline and proposed model is presented with the appropriate statistical significance.	☒	8	
**Model examination (Part 5)**	**Completed: page number**		**Notes if not completed**
Examination technique 1[a]	☒	19	
Examination technique 2[a]	☒	21 and 23	
A discussion of the relevance of the examination results with respect to model/algorithm performance is presented.	☒	10-11	
A discussion of the feasibility and significance of model interpretability at the case level if examination methods are uninterpretable is presented.	☒	9-11	
A discussion of the reliability and robustness of the model as the underlying data distribution shifts is included.	☒	10-11	
**Reproducibility (Part 6): choose appropriate tier of transparency**			**Notes**
Tier 1: complete sharing of the code		☒	
Tier 2: allow a third party to evaluate the code for accuracy/fairness; share the results of this evaluation		☐	
Tier 3: release of a virtual machine (binary) for running the code on new data without sharing its details		☐	
Tier 4: no sharing		☐	

PPV, positive predictive value; NNT, numbers needed to treat. [a]Common examination approaches based on study type: for studies involving exclusively structured data, coefficients and sensitivity analysis are often appropriate; for studies involving unstructured data in the domains of image analysis or natural language processing, saliency maps (or equivalents) and sensitivity analyses are often appropriate.

Supplementary Table 4. The MI-CLAIM (Minimum information about clinical artificial intelligence modeling) Checklist.

To maximize clinical safety and enable a direct and transparent assessment of the clinical impact of this work, we ensured adherence to established AI reporting guidelines (Norgeot, B., Quer, G., Beaulieu-Jones, B.K. et al. Minimum information about clinical artificial intelligence modeling: the MI-CLAIM checklist. Nat Med 26, 1320–1324 (2020). https://doi.org/10.1038/s41591-020-1041-y).

	Human	AI	Human-AI
**Precision**	0.3	0.41	0.93
**Recall**	0.33	0.50	0.92
**F1-Score**	0.31	0.43	0.92
**Accuracy**	0.53	0.73	0.93

Supplementary Table 5. Point estimates of overall performance metrics for the human rater, AI, and the Human-AI collaboration.

Values represent the point estimates for overall accuracy (proportion of correctly predicted cases), precision, recall, and F1-scores are summarized as macro-averages across all classes. Abbreviations: gross-total resection (GTR), near-total resection (NTR), subtotal resection (STR).

Variable	Aachen	Bielefeld	Hamburg	Denver (USA)	Cologne	Zurich (Switzerland)	Global p
Age	63.0 [55.0, 70.5]	63.0 [53.0, 72.0]	67.0 [59.0, 73.0]	62.0 [56.2, 69.0]	59.0 [51.8, 67.0]	63.2 [54.0, 71.7]	0.012
Year of surgery	2016.0 [2013.0, 2017.5]	2016.0 [2015.0, 2018.0]	2014.0 [2013.0, 2014.0]	2018.0 [2017.0, 2019.0]	2018.5 [2017.0, 2019.0]	2014.0 [2013.0, 2014.0]	<0.001
Tumor volume	29.6 [9.3, 59.2]	26.2 [10.0, 53.9]	31.9 [16.1, 56.4]	26.0 [13.0, 41.5]	15.6 [3.5, 37.5]	33.6 [14.1, 55.1]	0.023
Sex	151 (60.2%)	96 (55.2%)	77 (55.0%)	36 (72.0%)	35 (58.3%)	65 (65.0%)	0.214
Epilepsy	65 (26.0%)	56 (32.6%)	29 (20.9%)	17 (34.0%)	20 (33.3%)	32 (32.0%)	0.374
Frontal	102 (40.8%)	74 (42.5%)	43 (30.9%)	28 (56.0%)	19 (31.7%)	27 (27.0%)	0.023
Parietal	67 (26.8%)	63 (36.2%)	15 (10.7%)	18 (36.0%)	11 (18.3%)	33 (33.0%)	<0.001
Temporal	113 (45.2%)	98 (56.3%)	41 (29.7%)	16 (32.0%)	32 (53.3%)	46 (46.0%)	<0.001
Occipital	48 (19.2%)	30 (17.2%)	16 (11.6%)	6 (12.0%)	3 (5.0%)	11 (11.0%)	0.068
Insular	32 (12.8%)	52 (29.9%)	11 (8.0%)	6 (12.0%)	5 (8.3%)	23 (23.0%)	<0.001
Corpus callosum	42 (16.8%)	36 (20.7%)	34 (24.6%)	17 (34.0%)	6 (10.2%)	23 (23.0%)	0.041
Ventricle	85 (34.0%)	101 (58.0%)	4 (2.9%)	18 (36.0%)	21 (35.0%)	35 (35.0%)	<0.001
Midbrain	10 (4.0%)	2 (1.1%)	9 (6.5%)	1 (2.0%)	2 (3.3%)	0 (0.0%)	0.074
Eloquence	143 (57.2%)	104 (60.1%)	24 (17.4%)	25 (50.0%)	36 (60.0%)	47 (47.0%)	<0.001
Crossing midlines	42 (17.6%)	17 (9.8%)	12 (8.7%)	7 (14.0%)	2 (3.3%)	12 (12.0%)	<0.001
5-ala	57 (23.7%)	88 (50.6%)	52 (39.1%)	40 (80.0%)	60 (100.0%)	21 (21.0%)	<0.001
IONM	105 (43.8%)	41 (23.7%)	41 (30.6%)	30 (60.0%)	17 (28.3%)	14 (14.0%)	<0.001
Awake surgery	12 (4.9%)	2 (1.2%)	0 (0.0%)	20 (40.0%)	4 (6.7%)	0 (0.0%)	<0.001
Ultrasound	0 (0.0%)	1 (0.6%)	139 (100.0%)	2 (4.0%)	18 (30.0%)	92 (92.0%)	<0.001
Both hemispheres	33 (13.8%)	19 (10.9%)	13 (9.4%)	5 (10.0%)	1 (1.7%)	13 (13.0%)	0.002
Right	100 (41.8%)	80 (46.0%)	60 (43.5%)	24 (48.0%)	20 (33.3%)	46 (46.0%)	0.009
Left	106 (44.4%)	75 (43.1%)	65 (47.1%)	21 (42.0%)	39 (65.0%)	41 (41.0%)	<0.001
KPS	80.0 (54)	100.0 (52)	NA	80.0 (20)	90.0 (24)	80.0 (46)	<0.001
NANO	1.0 (57)	0.0 (45)	2.0 (47)	2.0 (14)	1.0 (24)	0.0 (22)	<0.001

Supplementary Table 6. Baseline clinical and tumor characteristics stratified by center.

Continuous variables are summarized as median [IQR] and compared using the Kruskal–Wallis test. Binary and categorical variables are summarized as counts (percentages) and compared using the χ^2 test (or Fisher's exact test where appropriate). Reported p-values reflect global differences across centers.

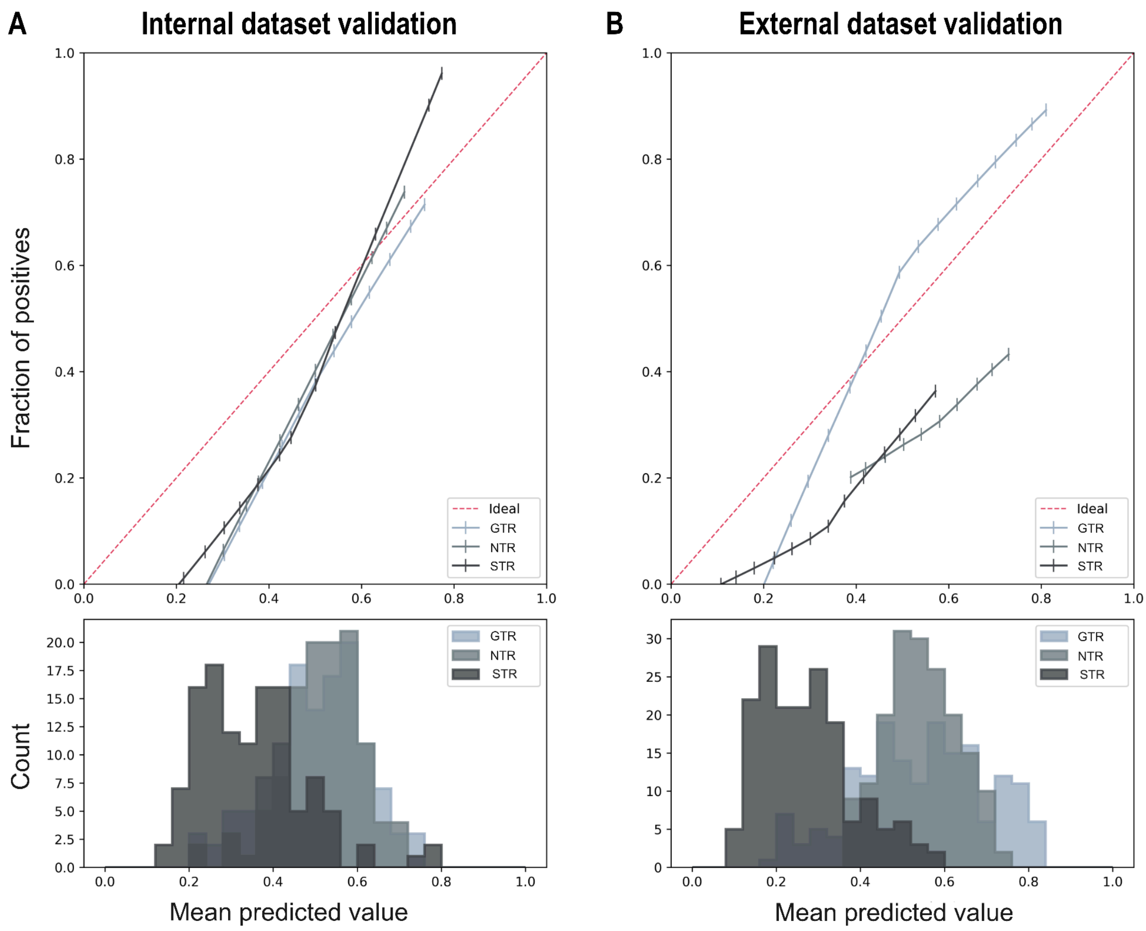

Supplementary Figure 1. Model calibration for internal and external validation.

Model calibration is visually assessed using calibration curves separately for (**A**) internal and (**B**) external validation. Perfect calibration corresponds to the ideal red line. Corresponding histograms are shown below. The quantitative assessment demonstrated Brier scores (ranges from 0 – 1; a score of 0 indicates perfect model calibration, and a score of 1 corresponds to poor model calibration) of 0.3 (GTR), 0.28 (NTR), and 0.44 (STR) for the internal validation cohort. In the external validation, Brier scores of 0.35 (GTR), 0.25 (NTR), and 0.52 (STR) demonstrated an overall well-calibrated multiclass model, indicating that the predicted EOR was in agreement with the observed EOR extent. Abbreviations: gross-total resection (GTR), near-total resection (NTR), subtotal resection (STR).

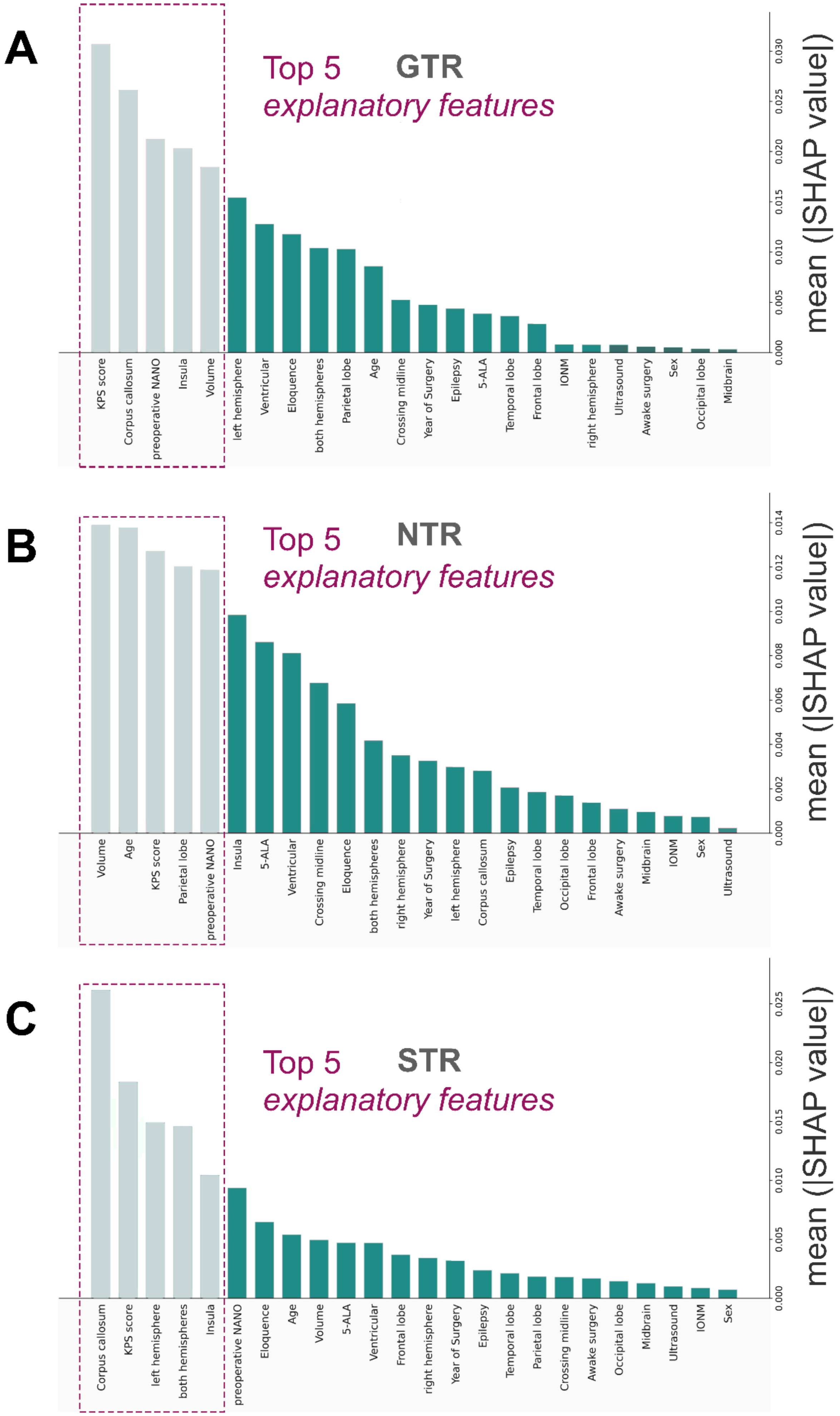
A
Top 5 GTR
explanatory features
KPS score
Corpus callosum
preoperative NANO
Insula
Volume
left hemisphere
Ventricular
Eloquence
both hemispheres
Parietal lobe
Age
Crossing midline
Year of Surgery
Epilepsy
5-ALA
Temporal lobe
Frontal lobe
IONM
right hemisphere
Ultrasound
Awake surgery
Sex
Occipital lobe
Midbrain
0.000
0.005
0.010
0.015
0.020
0.025
0.030
mean (|SHAP value|)
B
Top 5 NTR
explanatory features
Volume
Age
KPS score
Parietal lobe
preoperative NANO
Insula
5-ALA
Ventricular
Crossing midline
Eloquence
both hemispheres
right hemisphere
Year of Surgery
left hemisphere
Corpus callosum
Epilepsy
Temporal lobe
Occipital lobe
Frontal lobe
Awake surgery
Midbrain
IONM
Sex
Ultrasound
0.000
0.002
0.004
0.006
0.008
0.010
0.012
0.014
mean (|SHAP value|)
C
Top 5 STR
explanatory features
Corpus callosum
KPS score
left hemisphere
both hemispheres
Insula
preoperative NANO
Eloquence
Age
Volume
5-ALA
Ventricular
Frontal lobe
right hemisphere
Year of Surgery
Epilepsy
Temporal lobe
Parietal lobe
Crossing midline
Awake surgery
Occipital lobe
Midbrain
Ultrasound
IONM
Sex
0.000
0.005
0.010
0.015
0.020
0.025
mean (|SHAP value|)

Supplementary Figure 2. Global feature importance for each class prediction.

The explainability of the AI model is assessed on a *global level*, that is, separately for each class prediction of either GTR (**A**), NTR (**B**), or STR (**C**) across all patients. The bar plots display the absolute mean SHAP values (y-axis), where higher values correspond to a higher importance of the particular feature in facilitating an accurate class prediction. The corresponding *five most explanatory features* for each class prediction are marked in purple. Abbreviations: gross-total resection (GTR), near-total resection (NTR), subtotal resection (STR).

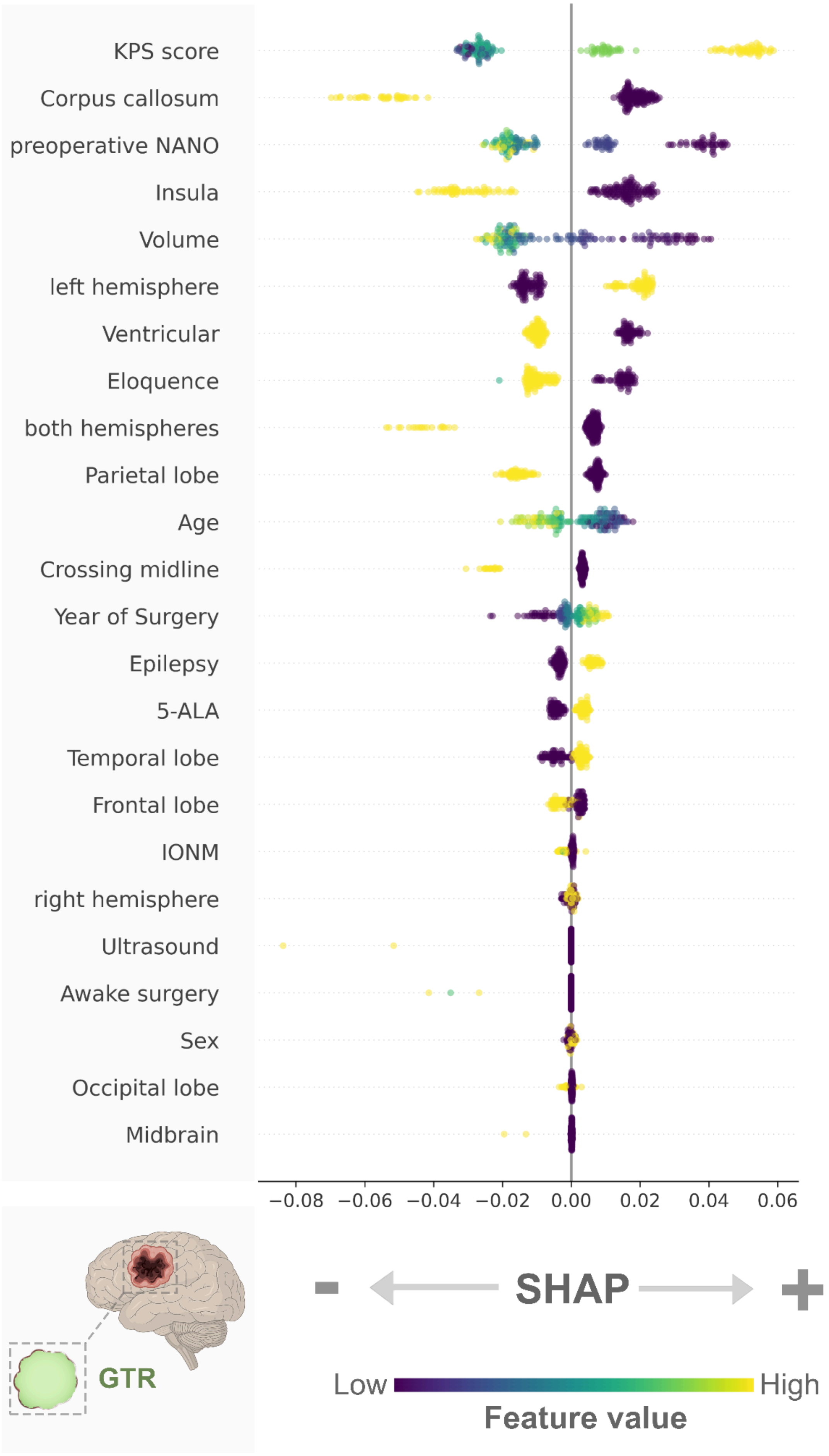
KPS score
Corpus callosum
preoperative NANO
Insula
Volume
left hemisphere
Ventricular
Eloquence
both hemispheres
Parietal lobe
Age
Crossing midline
Year of Surgery
Epilepsy
5-ALA
Temporal lobe
Frontal lobe
IONM
right hemisphere
Ultrasound
Awake surgery
Sex
Occipital lobe
Midbrain
−0.08 −0.06 −0.04 −0.02 0.00 0.02 0.04 0.06
GTR
SHAP
Low
High
Feature value

Supplementary Figure 3. SHAP explanation of predicting GTR.

The SHAP approach provides an excellent tool for interpreting feature effects. All features are ranked in descending order based on absolute explanatory importance (cf. **Suppl. Figure 2**). SHAP further allows for interpreting each feature's impact and direction on the prediction outcome. A positive SHAP value (x-axis) pushes the prediction towards the respective class outcome, e.g., predicting GTR. Additionally, the feature value (viridis color scale) must also be considered to interpret each feature effect correctly. As an example of interpreting feature effects, a high KPS score (yellow) impacts the model to predict GTR (positive SHAP value).

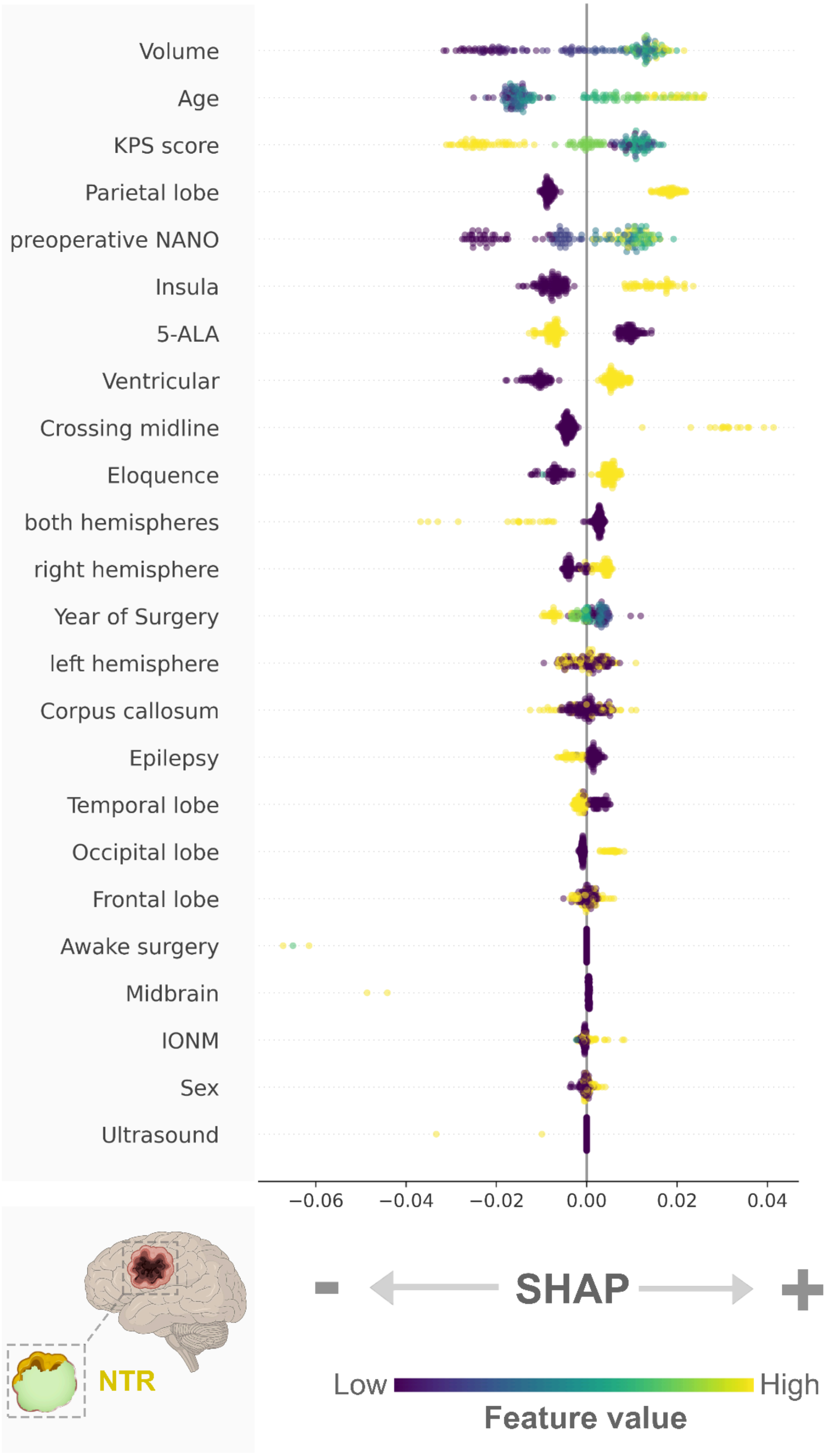
Volume
Age
KPS score
Parietal lobe
preoperative NANO
Insula
5-ALA
Ventricular
Crossing midline
Eloquence
both hemispheres
right hemisphere
Year of Surgery
left hemisphere
Corpus callosum
Epilepsy
Temporal lobe
Occipital lobe
Frontal lobe
Awake surgery
Midbrain
IONM
Sex
Ultrasound
−0.06
−0.04
−0.02
0.00
0.02
0.04
NTR
-
SHAP
+
Low
High
Feature value

Supplementary Figure 4. SHAP explanation of predicting NTR.

The SHAP approach provides an excellent tool for interpreting feature effects. All features are ranked in descending order based on absolute explanatory importance (cf. **Suppl. Figure 2**). SHAP further allows for interpreting each feature's impact and direction on the prediction outcome. A positive SHAP value (x-axis) pushes the prediction towards the respective class outcome, e.g., predicting NTR. Additionally, the feature value (viridis color scale) must also be considered to interpret each feature effect correctly. As an example of interpreting feature effects, a high tumor volume (yellow) impacts the model to predict NTR (positive SHAP value).

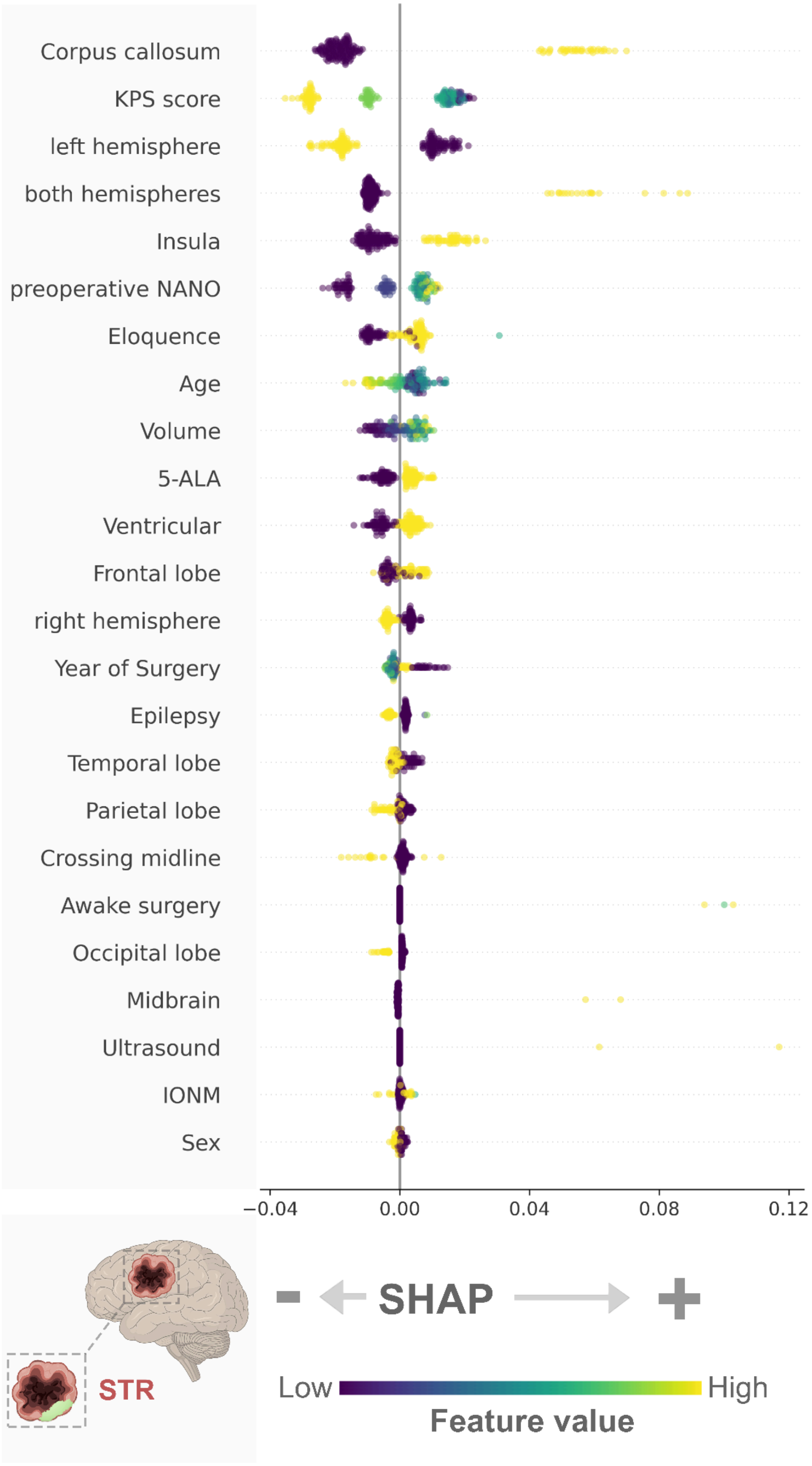

Corpus callosum
KPS score
left hemisphere
both hemispheres
Insula
preoperative NANO
Eloquence
Age
Volume
5-ALA
Ventricular
Frontal lobe
right hemisphere
Year of Surgery
Epilepsy
Temporal lobe
Parietal lobe
Crossing midline
Awake surgery
Occipital lobe
Midbrain
Ultrasound
IONM
Sex
−0.04
0.00
0.04
0.08
0.12
STR
SHAP
Low
High
Feature value

Supplementary Figure 5. SHAP explanation of predicting STR.

The SHAP approach provides an excellent tool for interpreting feature effects. All features are ranked in descending order based on absolute explanatory importance (cf. **Suppl. Figure 2**). SHAP further allows for interpreting each feature's impact and direction on the prediction outcome. A positive SHAP value (x-axis) pushes the prediction towards the respective class outcome, e.g., predicting STR. Additionally, the feature value (viridis color scale) must also be considered to interpret each feature effect correctly. As an example of interpreting feature effects, the corpus callosum infiltration (yellow) impacts the model to predict STR (positive SHAP value).

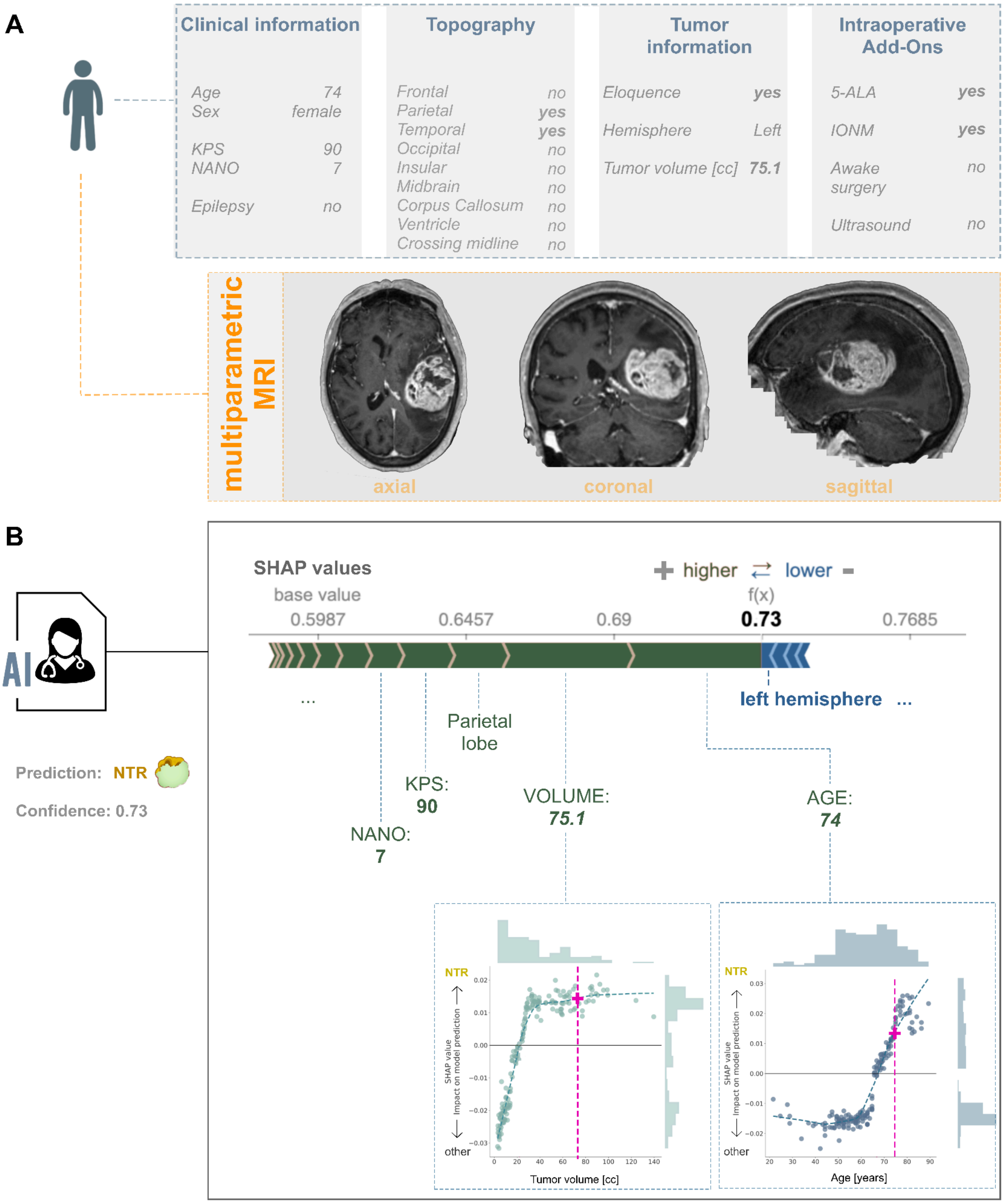

Supplementary Figure 6. Example of individual risk stratification using the *Human-AI collaboration* framework.

Panel (**A**) displays the information available to the human expert rater, including baseline clinical information, lesion topography, volumetric measures, supporting intraoperative efforts, and multiparametric MRI data. Panel (**B**) illustrates the Human-AI interface: the rater was additionally provided with the AI model's predicted resection class (e.g., NTR) and associated confidence score (e.g., 0.73), alongside SHAP-based model explanations visualizing the relative influence of clinical

and anatomical features on the prediction. To visualize and understand the model's rationale, we leveraged patient-specific SHAP force plots, where features contributing positively to the predicted class (e.g., NTR) are depicted in green (higher SHAP value, +), and features contributing negatively are visualized in blue (lower SHAP value, -). Additionally, individual feature thresholds, such as age and tumor volume, were visualized to contextualize each case (cf. **Figure 4** for thresholds). For example, the advanced patient's age of 74 (pink vertical line) surpasses the threshold of 67 years and thus contributes significantly to the predicted class. Likewise, a tumor volume exceeding 22 cc, which, as seen in this case at 75.1 cc, markedly exceeds the respective threshold and also adds to the prediction. This structured and interpretable interface enabled the human rater to understand and, where appropriate, integrate the AI model's reasoning into their clinical judgment for each case, therefore, facilitating a more informed and individualized reassessment process. In this collaborative workflow, clinicians used the AI output not as a final decision but as an augmentative layer, cross-referencing model insights with their expertise and contextual knowledge to reassess the likely EOR.

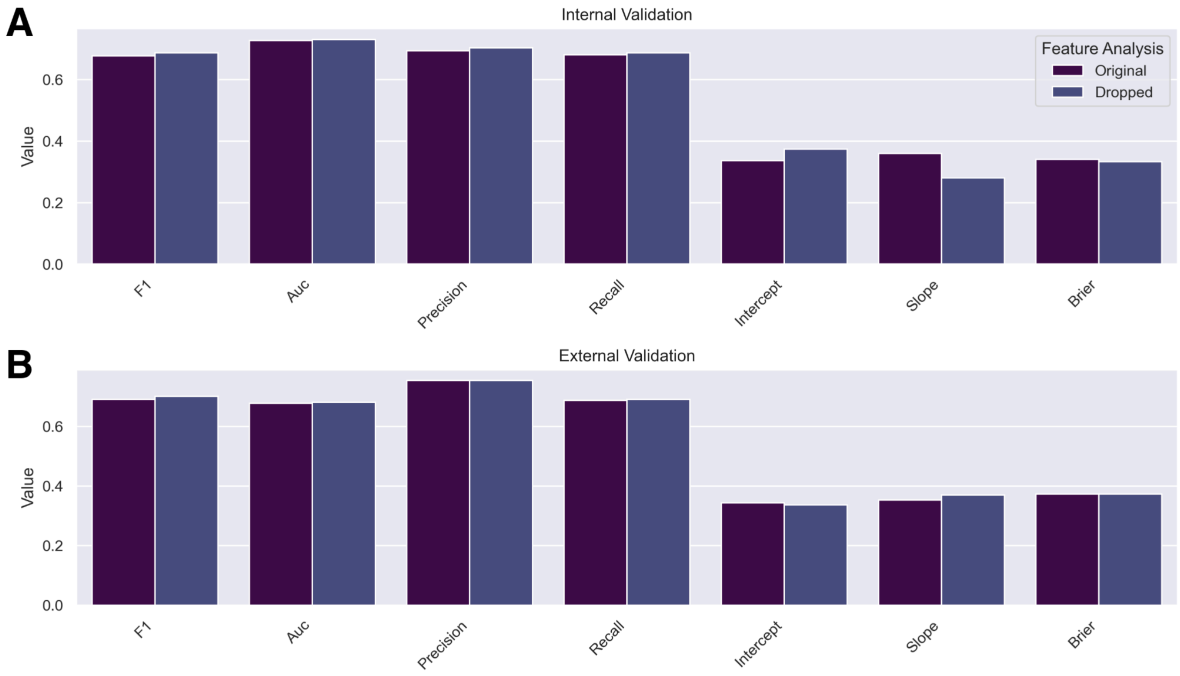

Supplementary Figure 7. Sensitivity analysis for the feature "Awake Surgery".

Bar plots comparing discrimination and calibration performance metrics across the same model trained with the imputed feature set (Original) and without the feature "Awake Surgery" (Dropped). Metrics are shown separately for the (**A**) internal and (**B**) external validation cohorts. Key metrics include F1 score, AUC, precision, recall, calibration intercept and slope, and Brier score. The minimal differences across all metrics support that in- or excluding "Awake Surgery" does not meaningfully affect model performance.

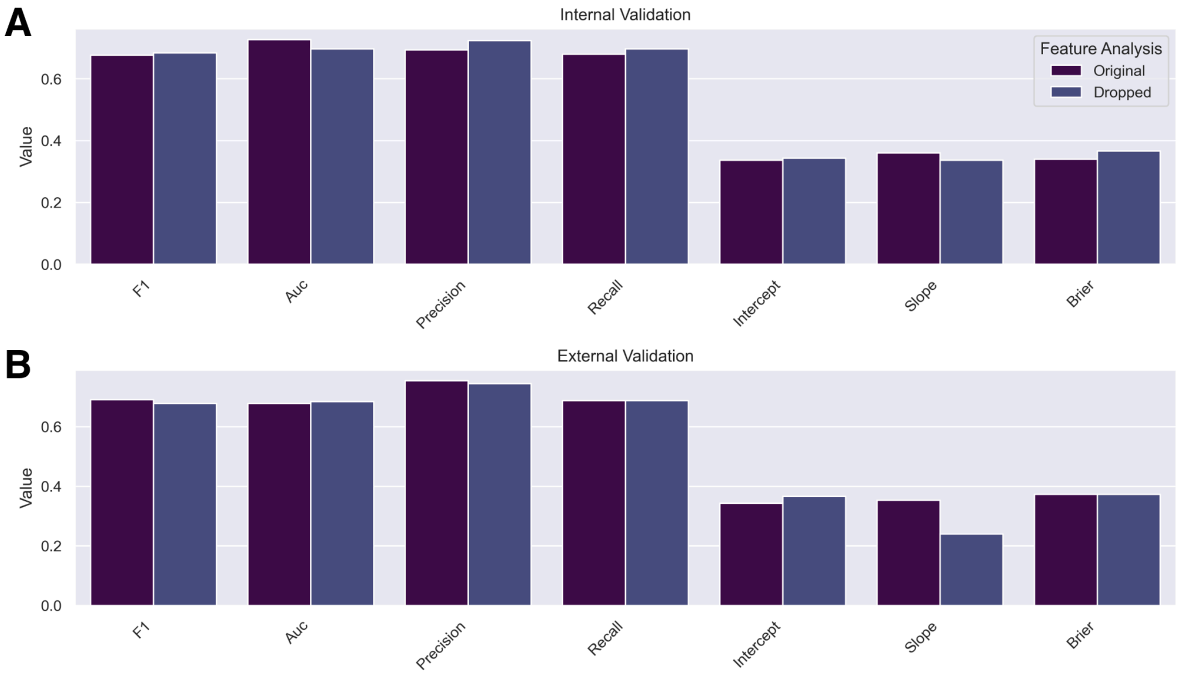

Supplementary Figure 8. Sensitivity analysis for the feature "Volume".

Bar plots comparing discrimination and calibration performance metrics across the same model trained with the imputed feature set (Original) and with all cases with missingness for "Volume" excluded (Dropped). Metrics are shown separately for the (**A**) internal and (**B**) external validation cohorts. Key metrics include F1 score, AUC, precision, recall, calibration intercept and slope, and Brier score. The minimal differences across all metrics support that the imputation of "Volume" does not meaningfully distort model performance.

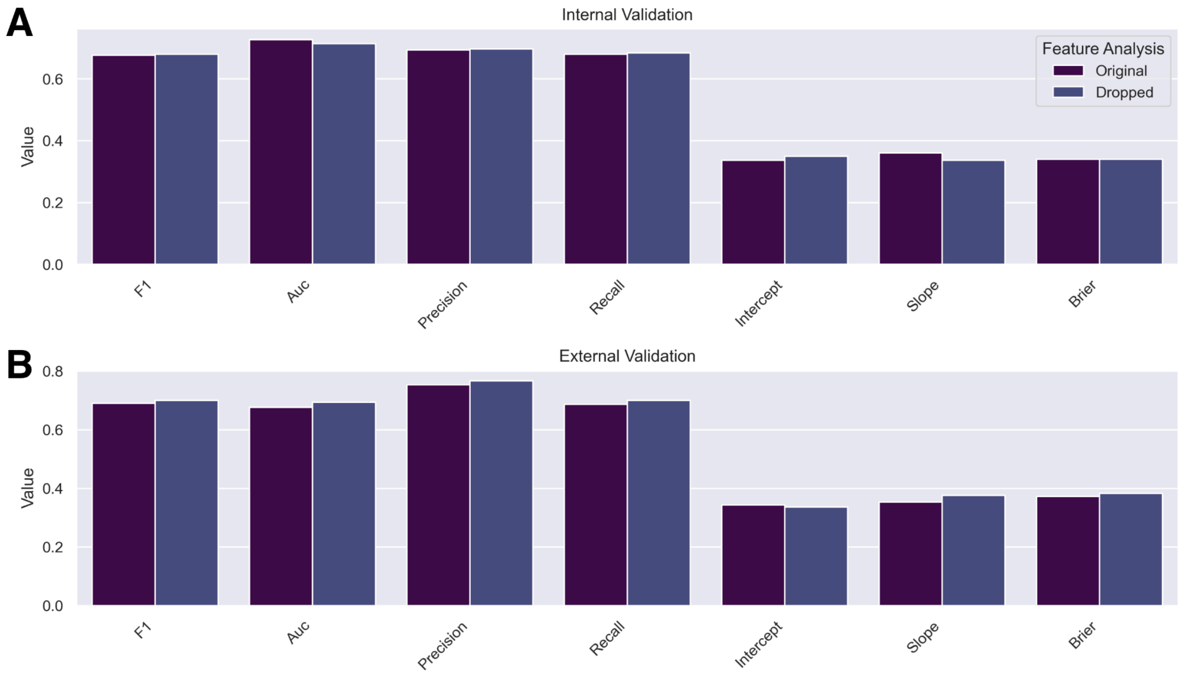

Supplementary Figure 9. Sensitivity analysis for features describing anatomical tumor location.

Bar plots comparing discrimination and calibration performance metrics across the same model trained with the imputed feature set (Original) and with all cases with missingness for any "Location" feature excluded (Dropped). Metrics are shown separately for the (**A**) internal and (**B**) external validation cohorts. Key metrics include F1 score, AUC, precision, recall, calibration intercept and slope, and Brier score. The minimal differences across all metrics support that the imputation of tumor "Locations" does not meaningfully distort model performance.

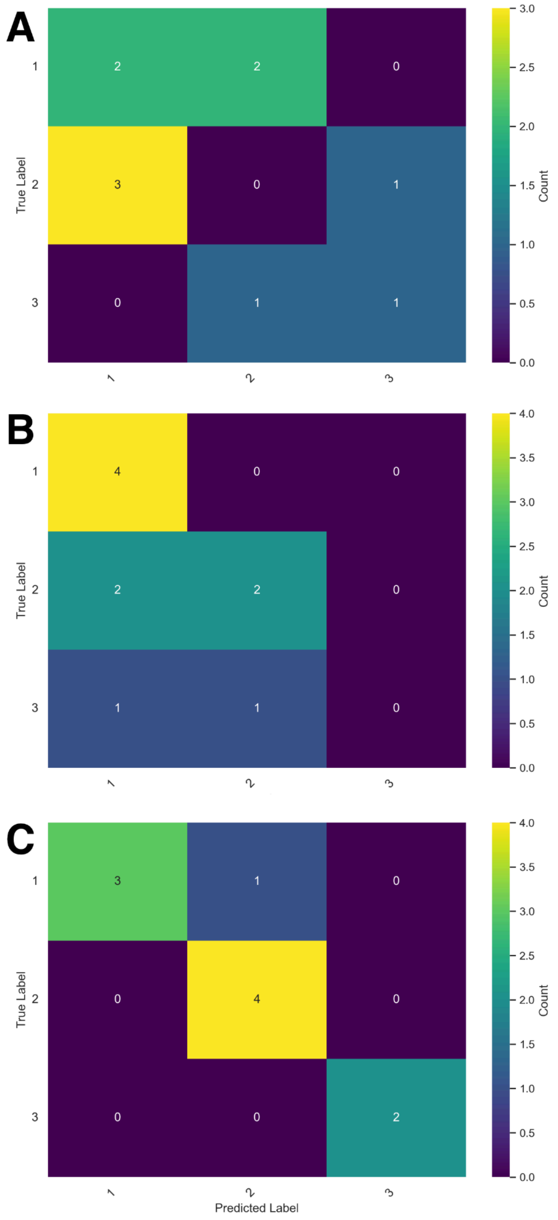

Supplementary Figure 10. Confusion matrices for human-alone, AI-alone, and the human-AI collaboration.

Shown separately for (**A**) human rater alone, (**B**) AI model alone, and (**C**) combined human-AI collaboration. Rows correspond to the true EOR class (1 = GTR, 2 = NTR, 3 = STR) and columns to the predicted class. Cell values indicate the count of cases in each true–predicted pairing. Notably, the human-AI collaboration panel (**C**) shows improved alignment along the diagonal compared to either human or AI alone.

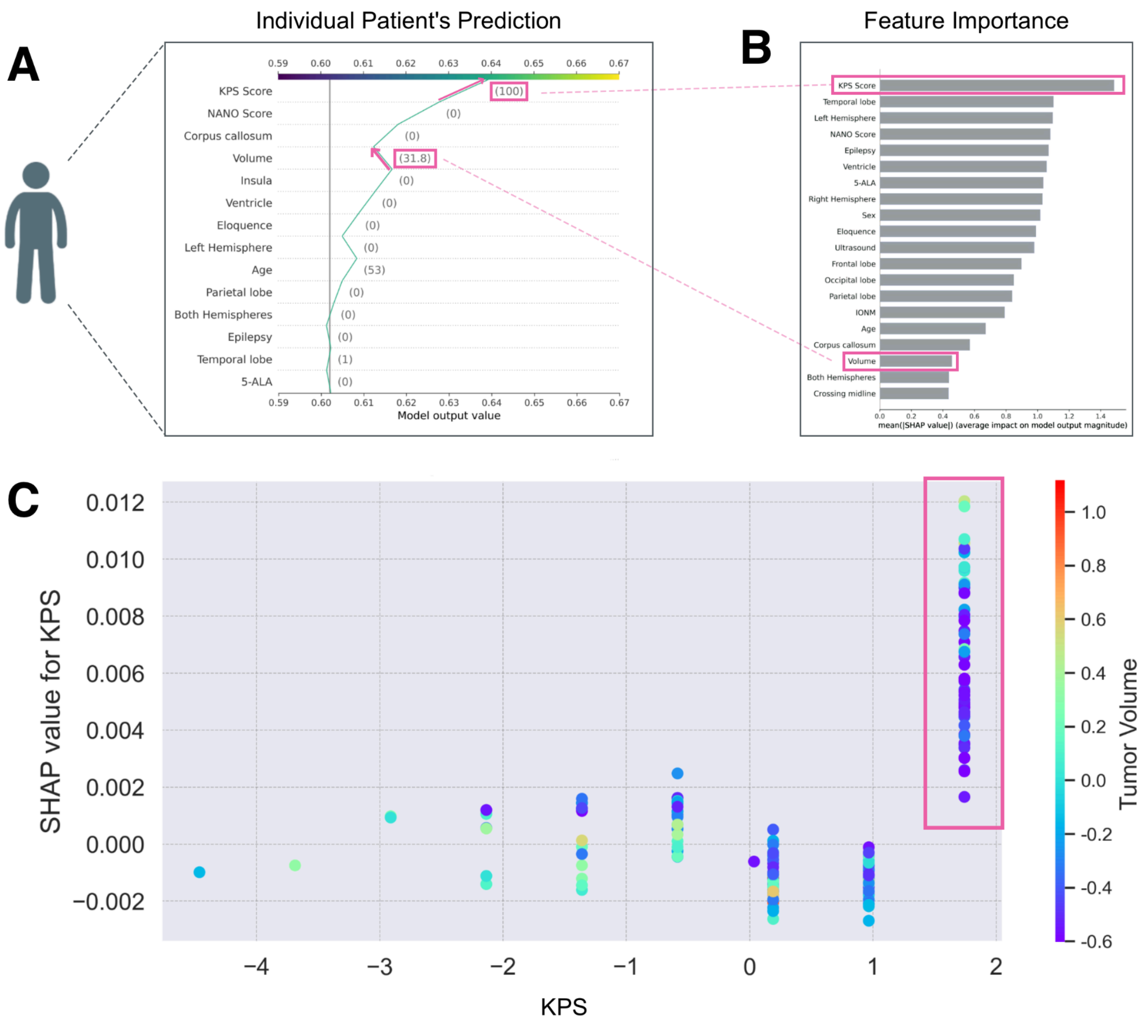

Supplementary Figure 11. Examination of high-confidence errors.

Panel (**A**) shows a SHAP decision plot for a true NTR case that was (incorrectly) predicted as GTR. The vertical gray line marks the model's baseline (≈0.60) before seeing any inputs. Each step in the plotted line represents a feature's contribution to the final GTR probability: importantly, the high KPS Score (100) marks the most positive "push" towards predicting GTR, while volume only marginally pulls the prediction back to "not GTR', even though tumor volume is large (~32 cc, cf. Figure 4 volume threshold for predicting NTR/STR between 22 - 25 cc). (**B**) The corresponding global feature‑importance across most confident but incorrect cases confirms that KPS has a substantially larger impact on the model's output than tumor volume. (**C**) SHAP dependence plot for the NTR class showing the relationship between KPS (x-axis) and its SHAP value (y-axis), colored by tumor volume. The densely packed points at high KPS (pink box) with high SHAP impact include cases with low tumor volume and large volume, where the model then fails to offset the functional‑status signal, illustrating the model's overreliance on KPS in these misclassified examples.

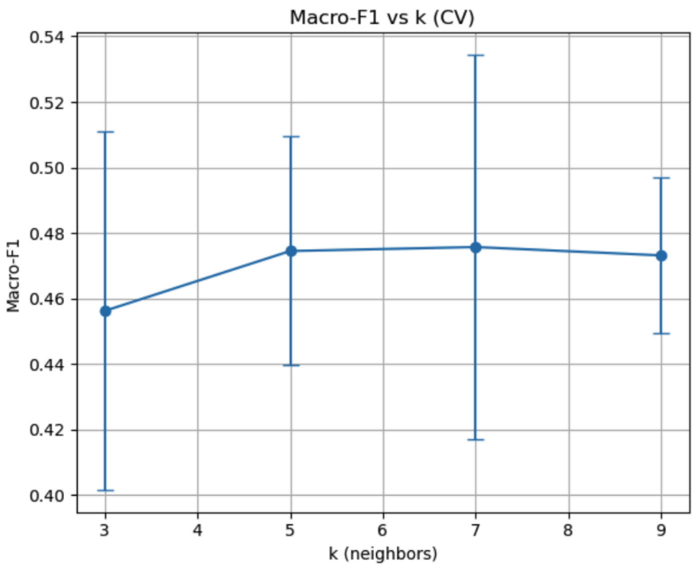

Supplementary Figure 12. Performance stability across different k values in KNN imputation.

Mean macro-F1 from 5-fold stratified cross-validation of a random forest classifier after imputing missing values with k = 3, 5, 7, and 9 using KNN imputation. The imputer was fit within each training fold and applied to the corresponding test fold to avoid data leakage. Error bars indicate the standard deviation across folds. While k = 7 and k = 5 achieved comparable mean performance, k = 5 showed the lowest variability, making it the most competitive and stable choice. These results confirm that the adopted setting of k = 5 provides a balance between predictive accuracy and robustness.

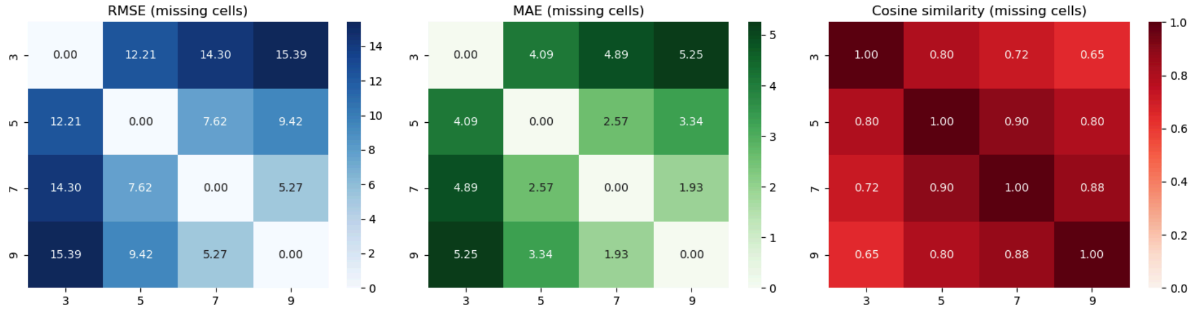

Supplementary Figure 13. Similarity of imputations across different k values.

Heatmaps show pairwise similarity between imputations obtained with k = 3, 5, 7, and 9, measured as RMSE (left) and MAE (middle) at missing cells, and cosine similarity after column-wise standardization (right). Imputations from k = 5 were consistently closest to both smaller (k = 3) and larger (k = 7, 9) neighbor choices, with the highest cosine similarity values (≥0.80). This confirms that k = 5 lies at the center of stable imputation behavior, supporting its use as the most robust choice.
